# Physiological and comparative proteome analyses reveal low-phosphate tolerance and enhanced photosynthesis in a maize mutant owing to reinforced inorganic phosphate recycling

**DOI:** 10.1186/s12870-016-0825-1

**Published:** 2016-06-08

**Authors:** Kewei Zhang, Hanhan Liu, Jiuling Song, Wei Wu, Kunpeng Li, Juren Zhang

**Affiliations:** Key Laboratory of Plant Cell Engineering and Germplasm Innovation, Ministry of Education, School of Life Science, Shandong University, 27 Shanda South Road, Jinan, 250100 People’s Republic of China; State Key Laboratory of Plant Physiology and Biochemistry, China Agricultural University, 17 Tsinghuadong Road, Beijing, 100083 People’s Republic of China

**Keywords:** Maize, Inorganic phosphorus, Low-phosphate-tolerant, Proteome, Leaf

## Abstract

**Background:**

The low-phosphate-tolerant maize mutant Qi319-96 was obtained from Qi319 through cellular engineering. To elucidate the molecular mechanisms underlying the low-phosphate tolerance of this mutant, we performed comparative proteome analyses of the leaves of Qi319-96 and Qi319 under inorganic phosphate (Pi)-sufficient and Pi-deficient conditions.

**Results:**

Low-phosphorus levels limit plant growth and metabolism. Although the overall phosphorus contents of shoots were not significantly different between Qi319 and Qi319-96, the Pi level of Qi319-96 was 52.94 % higher than that of Qi319. Under low phosphorus conditions, Qi319-96 had increased chlorophyll levels and enhanced photosynthesis. The changes in starch and sucrose contents under these conditions also differed between genotypes. The proteomic changes included 29 (Pi-sufficient) and 71 (Pi-deficient) differentially expressed proteins involved in numerous metabolic processes. Proteome and physiological analyses revealed that Qi319-96 could better remodel the lipid composition of membranes and had higher V-ATPase activity levels than Qi319 under low-phosphate starvation, which enhanced the recycling of intracellular Pi, as reflected by its increased Pi levels. Chlorophyll biosynthesis was improved and the levels, and activities, of several Calvin cycle and “CO_2_ pump” enzymes were greater in Qi319-96 than in Qi319, which led to a higher rate of photosynthesis under low-phosphate stress in this line compared with in Qi319.

**Conclusions:**

Our results suggest that the increased tolerance of the maize mutant Qi319-96 to low-phosphate levels is owing to its ability to increase Pi availability. Additionally, inbred lines of maize with low-P-tolerant traits could be obtained effectively through cellular engineering.

**Electronic supplementary material:**

The online version of this article (doi:10.1186/s12870-016-0825-1) contains supplementary material, which is available to authorized users.

## Background

Phosphorus is a vital plant macronutrient, functioning as a component in essential biomolecules such as phospholipids and ATP. Inorganic phosphate (Pi) plays central roles in virtually all of the major metabolic processes in plants, particularly photosynthesis [1, 2]. To further increase crop yields will require improving photosynthesis [3]. Thus, the efficient use of phosphorus during photosynthesis is a potentially important determinant of crop growth and yield.

Plants have evolved a series of strategies to cope with inadequate phosphate conditions while maintaining a proper balance of internal phosphorus levels [4]. These adjustments include (1) reducing phosphorus consumption by the plant [5], (2) increasing plant internal phosphorus recycling [6], and (3) improving phosphorus use in metabolic pathways [7].

Physiological and molecular adaptations that improve the phosphorus use efficiency include accelerated leaf senescence combined with the redirection of resources to growing tissues, as well as changes to metabolic pathways, such as primary carbon metabolism and phospholipid metabolism [8]. The release of phosphorus from membrane phospholipids by lipid remodeling is an important mechanism used by plants to adapt to low-phosphate conditions [9–11]. Sulfoquinovosyl diglyceride (SQDG) is a non-phosphorus lipid associated with several protein complexes in photosynthetic membranes, such as chloroplasts CF_0_-CF_1_ of ATPase, light harvesting complex II-apoproteins, and D1/D2 heterodimers [12]. The glycerophosphodiester-mediated lipid metabolic pathway may be involved in phosphorus release from phospholipids under low-phosphate stress. Sulfolipids and galactolipids, rather than phospholipids, are the major lipids of the thylakoid membrane in plants subjected to phosphate-deficiency stress. Under these conditions, plants can replace the phospholipids in photosynthetic membranes with specific non-phosphorus lipids [13]. These changes prolong and enhance the productive use of phosphorus during photosynthesis. Starch accumulation in the shoots is another common reaction of all plants to long-term phosphate deficiency [2]. One of the effects associated with starch accumulation is the release of phosphorus from chloroplasts to the cytoplasm for phosphorus recycling [14]. The accumulation of starch in phosphate-deficient leaves may help maintain the phosphorus balance between the cytoplasm and chloroplasts [15].

Increasing phosphorus recycling and phosphorus release from the vacuole may increase the phosphorus use efficiency. The vacuole is an important organelle involved in maintaining cytoplasmic phosphorus homeostasis [14, 16]. Excess cellular phosphorus in the cytoplasm is stored in the vacuole and is used to buffer the phosphorus demands of the cytoplasm [7]. The influx of phosphorus into the vacuole moderates phosphorus fluctuations by controlling the external intake of phosphorus and influencing cell metabolism. Under phosphorus deficiency, the V-ATPase gene may improve the proton transport to maintain an electrochemical gradient across the tonoplast by increasing its expression level, thereby providing the required energy to facilitate phosphorus transport [17].

Previously, we obtained the low-phosphate-tolerant mutant Qi319-96 from Qi319 using cellular engineering, therefore, they have a common genetic background. A comparison of the low-phosphate responses in these two maize genotypes indicated that low-phosphate tolerance is greater in the Qi319-96 genotype than in Qi319. The light energy conversion efficiency and Pi contents are higher in Qi319-96 than in Qi319 under low-phosphate conditions [18]. We previously performed a systematic proteome analysis of Qi319 maize leaves in response to phosphate starvation, finding that the phosphate starvation response is a complicated process involving several metabolic reactions, such as photosynthesis, carbohydrate metabolism, energy metabolism, secondary metabolism, signal transduction, and protein synthesis. After being subjected to a long period of phosphorus stress, the internal phosphorus use efficiency in Qi319 maize may increase through altered photorespiration and lipid composition, along with increased starch synthesis [19]. To elucidate the molecular mechanisms of the different tolerance levels to low-P conditions between Qi319-96 and Qi319, a comparative proteome analysis should be performed.

In this study, we performed comparative proteome analyses of leaves from mutant Qi319-96 and wild-type Qi319 plants treated with 1000 μM (+P, Pi-sufficient) and 5 μM (–P, Pi-deficient) KH_2_PO_4_ over a long time period. The objectives of this study were (i) to determine the reasons behind the differences in leaf Pi levels between the two genotypes; (ii) to investigate the mechanism behind the high photosynthetic efficiency levels in Qi319-96; and (iii) to provide information for further research into the functions of genes involved in phosphate-stress responses.

## Results

### Differential growth and physiological responses to phosphate deprivation between Qi319 and Qi319-96

**Fig. 1:**
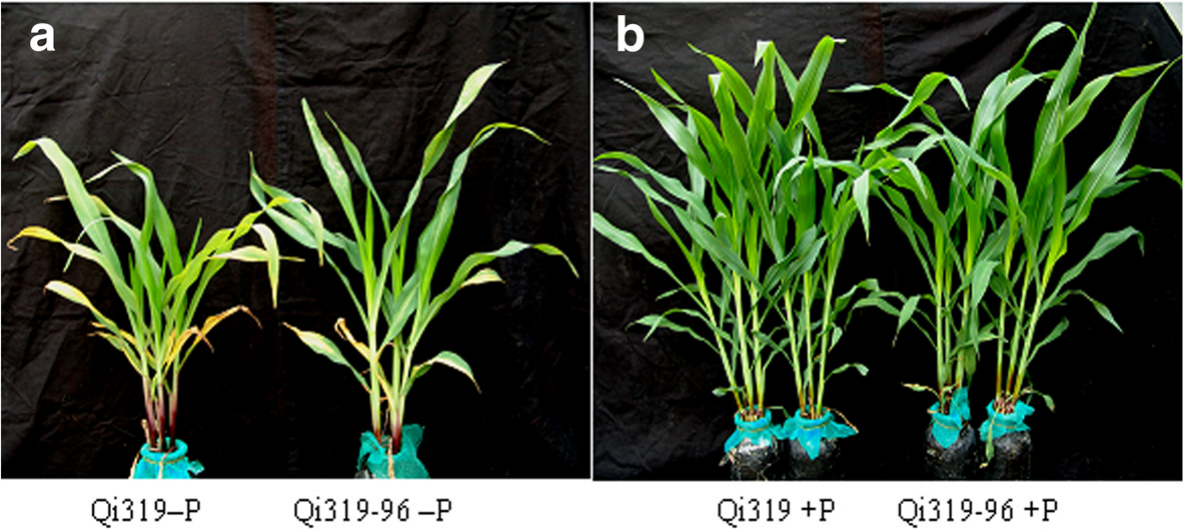
Qi319 and Qi319-96 maize plants grown in two growth conditions + P and -P. **a:** Qi319 and Qi319-96 grew under -P conditions (5 μmol KH_2_PO_4_). **b:** Qi319 and Qi319-96 grew under + P conditions (1000 μmol KH_2_PO_4_)

After treatment with 5 μmol KH_2_PO_4_ for 25 days, Qi319 and Qi319-96 maize plants grew to the six- to seven-leaf stage but exhibited apparent phosphorus deficiency symptoms, such as reduced overall phosphorus contents, marked changes in biomass (Table 1), heliotrope-colored stems, and restricted growth (Fig. 1).

**Table 1:** Influence of different phosphate treatments on biomass, inorganic phosphorus concentration and phosphorus contents of Qi319 and Qi319-96

Inbred lines	Treatment	Inorganic phosphorus content (μg g^-1^ FW)	Biomass (g/three plant)			P contents (mg P g^-1^DW)		
			Root	Shoot	total	Root	Shoot	total
Qi319-96	+P	24.29 ± 1.01a	4.36 ± 0.08a	8.70 ± 0.09a	13.06 ± 0.17a	1.71 ± 0.05a	2.56 ± 0.03a	2.27 ± 0.04a
	-P	17.39 ± 0.99c	4.01 ± 0.06b	5.65 ± 0.15b	9.66 ± 0.21b	0.58 ± 0.01b	0.87 ± 0.01b	0.75 ± 0.02b
Qi319	+P	20.22 ± 1.02b	4.47 ± 0.03a	8.84 ± 0.06a	13.31 ± 0.15a	1.61 ± 0.07a	2.48 ± 0.06a	2.19 ± 0.07a
	-P	11.37 ± 0.86d	3.35 ± 0.03c	5.37 ± 0.09b	8.72 ± 0.07c	0.44 ± 0.01c	0.86 ± 0.02b	0.68 ± 0.02c

Three seedlings per bottle were cultured in phosphorus saturation solution (1000 μM KH_2_PO_4_) to the three-leaf stage, followed by low phosphate stress solution (5 μM KH_2_PO_4_) for an additional 25 days to the six–seven-leaf stage. Values represent the means of nine seedlings ± SD. Values with different letters within a row are significantly different (*P* < 0.05) by multiple comparison analysis

The phosphorus contents, and root and shoot biomasses, were not significantly different between Qi319-96 and Qi319 under the sufficient phosphate (+P) treatment. However, the root biomasses were significantly higher in Qi319-96 than in Qi319 after phosphorus stress treatments. The phosphorus content in roots was significantly higher in Qi319-96 than in Qi319, while the overall phosphorus content of the shoots did not significantly differ between genotypes (Table 1). However, the Pi levels of Qi319-96 and Qi319 decreased by 28.41 % and 43.77 %, respectively, after the phosphorus deficiency treatment, but the Pi content in shoots was still 52.95 % higher in Qi319-96 than in Qi319 (Table 1).

Low-phosphate stress limits plant photosynthesis (Table 2). Under phosphate-deficiency conditions, the net photosynthesis rate (*P*n) decreased by 49.88 % in Qi319 and 41.33 % in Qi319-96. Under low-phosphate stress, the *P*n of Qi319-96 was 26.81 % higher than that of Qi319. Phosphate deficiency also reduced the stomatal limitation value (*L*s). The *L*s values of the two genotypes, when subjected to the sufficient phosphorus treatment, were not significantly different, but the intercellular CO_2_ concentration (*C*i) increased in both genotypes after low-phosphate stress (Table 2). The chlorophyll content in leaves was 50.00 % higher in Qi319-96 than in Qi319 (Table 3), indicating that photo-absorption by the chloroplasts was better in Qi319-96 under low-phosphate conditions.

**Table 2:** Influence of different phosphate treatments on photosynthesis in Qi319 and Qi319-96

Inbred lines	P treatment	*P*n (μmol CO_2_ m^-2^ s^-1^)	*C*i (μmol mol^-1^)	*L*s
Qi319-96	+P	22.09 ± 0.35a	76.49 ± 1.56c	0.79 ± 0.03a
	-P	12.96 ± 0.40c	108.51 ± 3.58b	0.69 ± 0.02b
Qi319	+P	20.39 ± 0.14b	71.21 ± 5.57c	0.80 ± 0.03a
	-P	10.22 ± 0.39d	143.06 ± 2.29a	0.60 ± 0.01c

Three seedlings per bottle were cultured in phosphorus saturation solution (1000 μM KH_2_PO_4_) to the three-leaf stage, followed by low phosphate stress solution (5 μM KH_2_PO_4_) for an additional 25 days to the six–seven-leaf stage. Values represent the means of nine seedlings ± SD. Values with different letters within a row are significantly different (*P* < 0.05) by multiple comparison analysis

**Table 3:** Influence of different phosphate treatments on sucrose, starch and chlorophyll contents

Inbred lines	P treatment	Sucrose content (mg g^-1^ DW)	Starch content (mg g^-1^ DW)	Chorophyll content (mg g^-1^ FW)
Qi319-96	+P	46.61 ± 1.22a	200.22 ± 1.45b	2.54 ± 0.18a
	-P	31.51 ± 1.32c	210.34 ± 1.56a	1.47 ± 0.16c
Qi319	+P	40.84 ± 1.33b	189.25 ± 1.48c	2.13 ± 0.15b
	-P	25.63 ± 1.24d	209.39 ± 1.55a	0.98 ± 0.09d

Three seedlings per bottle were cultured in phosphorus saturation solution (1000 μM KH_2_PO_4_) to the three-leaf stage, followed by low phosphate stress solution (5 μM KH_2_PO_4_) for an additional 25 days to the six–seven-leaf stage. Values represent the means of nine seedlings ± SD. Values with different letters within a row are significantly different (*P* < 0.05) by multiple comparison analysis

The sucrose contents in the leaves significantly declined under low-phosphate stress, but the starch contents were still higher than the contents detected under phosphate-sufficient conditions (Table 3). The sucrose contents in the leaves were higher in Qi319-96 than in Qi319. More of the photosynthetic products were used for sucrose biosynthesis in Qi319-96 than in Qi319. These data indicated that the distribution of the photosynthetic carbon metabolism between sucrose and starch was altered in both genotypes under low-phosphate conditions. Thus, the low-phosphate-tolerant mutant Qi319-96 had a higher photosynthetic CO_2_ fixation rate and plant biomass compared with wild-type Qi319. Although the phosphorus level in shoots did not differ between genotypes, Qi319-96 had significantly higher levels of Pi than Qi319.

### Differential analysis of leaf protein profiles

**Fig. 2:**
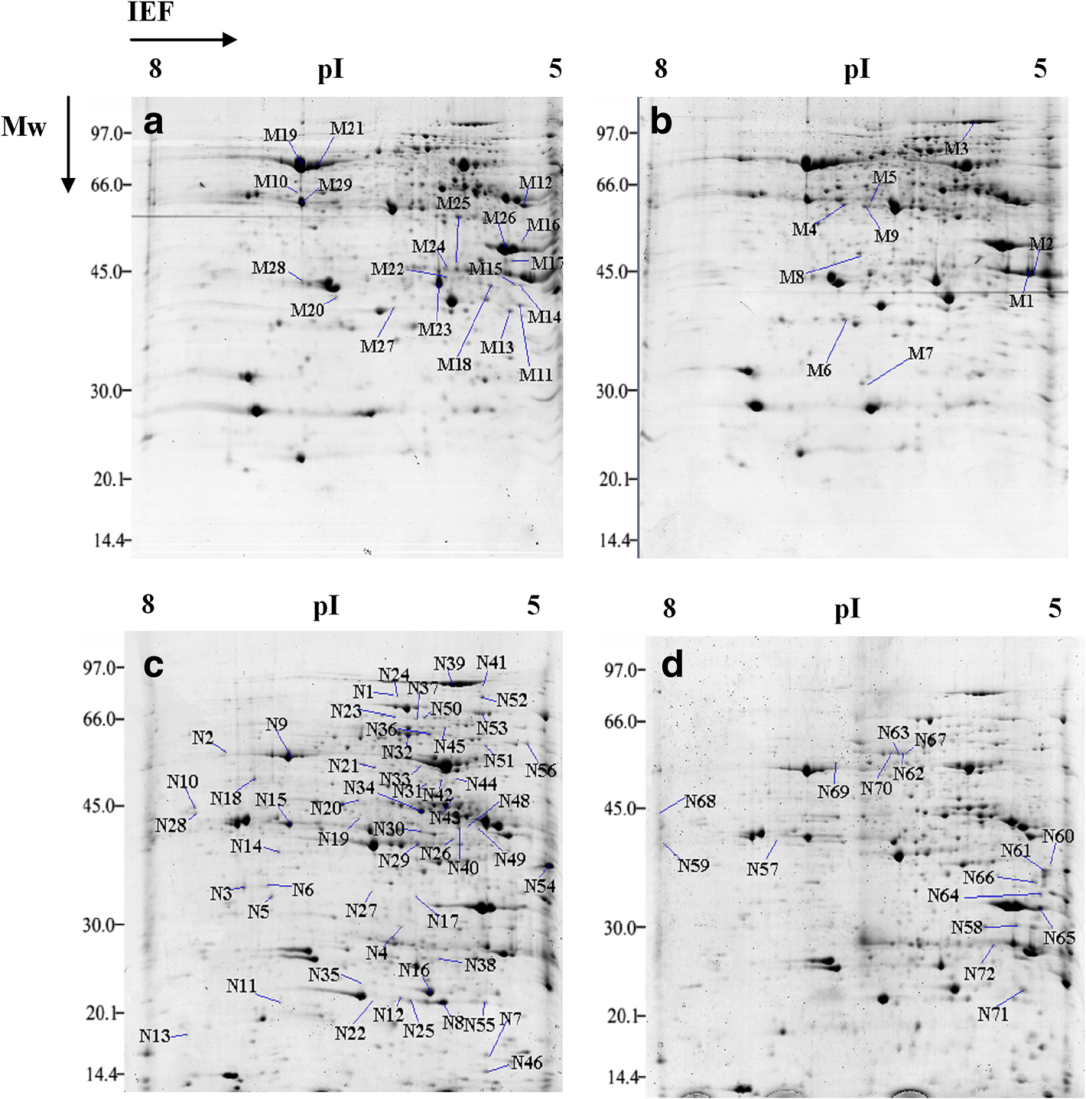
Comparison 2-DE protein gel maps taken from Qi319 and Qi319-96 maize leaves that had been subjected to two different phosphorus concentrations. The proteins were extracted by TCA/acetone from the middle of the fourth leaf and the 1.2 mg protein samples were separated in IEF using 17 cm pH 5–8 IPG strips. Then they were put on a 12 % polyacrylamide gel for the second dimensional separation and stained with CBB. The gel image analysis was carried out using PDQuest software (version 7.2.0; Bio-Rad). The spots marked with numbers (M1–M29) indicated proteins that were differentially accumulated in leaves of Qi319-96 and Qi-319 under + P conditions identified by MALDI-TOF MS; The spots marked with numbers (N1-N72) indicate proteins that were differentially accumulated in leaves of Qi319-96 and Qi-319 under -P conditions identified by MALDI-TOF MS. **a**: the image of the + P treatment Qi319-96 protein; **b**: the image of the + P treatment Qi319 protein; **c**: the image of the –P treatment Qi319-96 protein; **d**: the image of the –P treatment Qi319 protein

We performed comparative proteomic studies of Qi319 and Qi319-96 maize leaves subjected to two different phosphate levels using immobilized pH gradient (IPG) strips (pH 5–8), with three biological replicates. The number of protein spots detected in the gels and the proteins that differentially accumulated in the two genotypes are summarized in Table 4. Approximately 680 spots were detected under the phosphate saturation treatment. Of these, 29 (4.26 %) spots differentially accumulated between Qi319 and Qi319-96. Of the 29 spots, nine spots (including proteins not visible in the Qi319-96 gels) accumulated to a greater extent in Qi319 than in Qi319-96, whereas 20 spots (including proteins not visible in the Qi319 gels) accumulated to a greater extent in Qi319-96 (Fig. 2a, b). After the phosphate-deficiency treatment, 592 spots were detected, 71 (11.99 %) of which differentially accumulated between Qi319 and Qi319-96. Of these, 55 spots (including proteins not visible in the Qi319 gels) accumulated to a greater extent in Qi319-96 than in Qi319, whereas 16 spots (including proteins not visible in the Qi319-96 gels) accumulated to a greater extent in Qi319 (Fig. 2c, d).

**Table 4:** Number of differentially accumulated proteins between the two genotypes and in response to phosphate stress on 2-DE gels (Fig. 2)

	Qi319 + P versus Qi319-96 + P	Qi319 – P versus Qi319-96 – P
No. of proteins over-accumulated in Qi319-96	9	55
No. of proteins over-accumulated in Qi319	20	16
Total no. of differentially accumulated proteins	29	71

### Identification and classification of phosphate-stress-responsive proteins

We identified the proteins that were differentially expressed after the two phosphate treatments using matrix-assisted laser desorption/ionization tandem time-of-flight mass spectrometry (MALDI-TOF MS) to gain a better understanding of the mechanisms involved in phosphorus stress and the differences in phosphorus tolerance between Qi319-96 and Qi319 maize plants. In total, 100 proteins were identified using the NCBI databases (Table 5 and Additional file 1). The detailed peptide sequences are shown in Additional file 2. We classified these proteins based on the TAIR (http://www.arabidopsis.org/) and KEGG (http://www.genome.jp/kegg) databases. The proteins were classified into protein fate, protein synthesis, cell rescue/defense/virulence, metabolism, energy and transcription/signal transduction mechanisms (Table 5). To confirm the results produced by peptide mass finger printing (PMF), eight randomly selected spots from among these proteins were subjected to MALDI-TOF-TOF MS analysis. The results for all eight were consistent with the PMF results (Additional file 3 and Additional file 4), which confirmed their reliability.

**Table 5:** Differentially accumulated proteins with similar functionsin Qi319-96 and Qi319 leaves under both + P and − P conditions

Matched protein name^*a*^	gi Number^b^	Spot no.^*c*^	Pattern^*d*^	Qi319-96– P versus Qi319-96 + P^*e*^	Qi319– P versus Qi319 + P^*f*^
		Qi319-96 + P/Qi319 + P			
Metabolism					
Chlorophyll A-B binding protein	gi|242088861	M1	Increase	1:1.04	1.07:1
Chlorophyll A-B binding protein	gi|194700378	M2	Increase	1:1.70	1: 5.05
Chain A, Pyruvate phosphate dikinase	gi|62738111	M3	Increase	1.62:1	1.78:1
malate dehydrogenase	gi|226498728	M4	Increase	1:1.74	1:2.03
Coproporphyrinogen III oxidase	gi|308081534	M5	Increase	6.93:1	1:60.35
Triosephosphate isomerase	gi|194702698	M9	Increase	1.75:1	1:2.96
hydroxypyruvate reductase	gi|194697898	M10	Decrease	1.30:1	1.07:1
thylakoid lumenal 19 KDa protein	gi|226491484	M11	Decrease	4.89:1	2.72:1
sedoheptulose bisphosphatase1	gi|226506366	M12	Decrease	1:1.12	1:1.72
Cyanobacterial and plastid NDH-1 subunit M	gi|194701566	M13	Decrease	1:7.25	1:2.25
ribulose-1,5-bisphosphate carboxylase/oxygenase large subunit	gi|144583482	M19	Decrease	1:19.87	5.91:1
Ribulose bisphosphate carboxylase large chain	gi|132061	M20	Decrease	1:6.24	1:3.99
ribulose-1,5-bisphosphate carboxylase/oxygenase large subunit	gi|11467200	M21	Decrease	1:1.11	1:3.09
ribulose-1,5-bisphosphate carboxylase/oxygenase large subunit	gi|260677427	M22	Decrease	1.30:1	1.08:1
chlorophyll a-b binding protein 8	gi|195613254	M23	Decrease	1:3.41	1:2.12
oxygen-evolving enhancer protein 1	gi|195619530	M26	Decrease	1:2.45	1:4.89
NADH dehydrogenase subunit I	gi|11467259	M28	Decrease	1:8.84	1:1.71
Energy					
GlyoxalaseI	gi|212274373	M16	Decrease	1:1.27	8.09:1
glyceraldehyde-3-phosphate dehydrogenase A, chloroplastic	gi|162461856	M17	Decrease	1:1.49	1:1.39
GADPH (383 AA)	gi|22240	M29	Decrease	7.99:1	2.09:1
Protein fate					
peptidyl-prolyl cis-trans isomerase	gi|226491656	M6	Increase	1:18.53	1:35.85
chaperonin	gi|195623400	M14	Decrease	1:1.19	2.20:1
CPN10	gi|194688414	M15	Decrease	1:1.50	3.19:1
Pepsin-like aspartate proteases	gi|168041407	M24	Decrease	1:1.39	1:16.85
peptide methionine sulfoxide reductase	gi|226532399	M27	Decrease	4.14:1	3.16:1
Protein synthesis					
ribonucleoprotein	gi|22942613	M18	Decrease	2.47:1	1:17.3
Transcription/cellular communication/signal transduction					
glycine-rich RNA binding protein 2	gi|195609654	M7	Increase	1:10.72	1:15.2
Unknown					
hypothetical protein SORBIDRAFT_09g001130	gi|242086601	M8	Increase	4.21:1	1.17:1
LOC542632	gi|162462462	M25	Decrease	10.09:1	1.24:1
		Qi319-96 – P/Qi319 – P			
Metabolism					
ribulose-1,5-bisphosphate carboxylase/oxygenase large subunit	gi|144583490	N3	Increase	1:7.29	1:3.06
ribulose-1,5-bisphosphate carboxylase/oxygenase large subunit	gi|27448357	N4	Increase	1:1.35	1:2.99
ribulose-1,5-bisphosphate carboxylase/oxygenase large subunit	gi|290086785	N5	Increase	1:1.89	2.41:1
ribulose-1,5-bisphosphate carboxylase/oxygenase large subunit	gi|135991663	N6	Increase	1:6.23	1:1.87
ribulose-1,5-bisphosphate carboxylase/oxygenase large subunit	gi|3560858	N7	Increase	1.86:1	1:12.11
ribulose-1,5-bisphosphate carboxylase/oxygenase large subunit	gi|224382434	N8	Increase	4.21:1	6.33:1
ribulose-1,5-bisphosphate carboxylase/oxygenase large subunit	gi|11467200	N9	Increase	1:16.96	1:19.00
ribulose-1,5-bisphosphate carboxylase/oxygenase large subunit	gi|144583486	N11	Increase	1:4.14	1:3.37
Ribulose bisphosphate carboxylase large chain	gi|261279200	N12	Increase	1:4.78	1:6.44
ribulose-1,5-bisphosphate carboxylase/oxygenase large subunit	gi|164565217	N14	Increase	1:6.53	1:3.59
chlorophyll a-b binding protein 8	gi|226496924	N15	Increase	1:7.07	9.89:1
Chlorophyll a-b binding protein6A	gi|223973225	N16	Increase	1:1.33	1.33:1
chlorophyll a-b binding protein 8	gi|195613254	N17	Increase	3.79:1	1:10.14
UDP-sulfoquinovose synthase	gi|238014584	N18	Increase	4.69:1	3.63:1
NDH-dependent cyclic electron flow 1 NAD-dependent epimerase	gi|194704742	N19	Increase	1:1.33	1:2.93
sucrose-phosphatase 1	gi|194699500	N21	Increase	1:1.79	1:6.51
NADP-dependent malic enzyme,chloroplastic	gi|162459265	N32	Increase	1.27:1	1:5.15
NADP-dependent malic enzyme, chloroplastic precursor	gi|162459265	N35	Qi319-96 only	1:1.59	0:1
NADP-dependent malic enzyme, chloroplastic	gi|162459265	N36	Increase	1.05:1	1:8.35
pyruvate orthophosphate dikinase	gi|285013667	N38	Increase	2.24:1	4.35:1
pyruvateorthophosphate dikinase	gi|168586	N39	Increase	1:1.66	1:7.35
pyruvate orthophosphate dikinase	gi|219819651	N41	Qi319-96 only	1:2.26	0:1
delta-aminolevulinic acid dehydratase	gi|226496321	N43	Increase	1:2.07	1:2.96
transferase, transferring glycosyl groups, putative	gi|255562878	N44	Increase	1:4.52	1:6.58
glucose-6-phosphate isomerase	gi|226530882	N51	Increase	1:1.31	1:1.91
Low PSII Accumulation 3	gi|242060200	N54	Increase	2.07:1	2.59:1
subunit NDH-M of NAD(P)H:plastoquinone dehydrogenase	gi|226497418	N55	Increase	1:1.85	1.08:1
carbonate dehydratase	gi|293332983	N69	Decrease	1:2.58	1:3.07
thylakoid lumenal 19 kDa protein	gi|226491484	N71	Decrease	1:1.83	3.33:1
Energy					
NADP^+^-dependent non-phosphorylating glyceraldehyde-3-phosphate dehydrogenase B	gi|194688752	N1	Increase	1:1.72	1:1.98
NADP^+^-dependent non-phosphorylating glyceraldehyde-3-phosphate dehydrogenase B	gi|194700892	N2	Increase	2.04:1	1:1.68
6-phosphogluconate dehydrogenase family protein	gi|162463282	N10	Increase	1.99:1	1:1.50
alcohol dehydrogenase 2	gi|195613268	N20	Increase	1:1.02	1:1.68
phosphoglucomutase, cytoplasmic 1	gi|162463106	N23	Increase	2.21:1	1:3.65
fructose-bisphosphate aldolase	gi|195622374	N26	Increase	8.56:1	1:4.94
fructose-bisphosphate aldolase	gi|223975775	N27	Increase	1:4.39	1:3.22
fructose-bisphosphate aldolase	gi|194690156	N28	Increase	2.90:1	2.18:1
fructose-bisphosphate aldolase	gi|195634659	N29	Increase	1.41:1	1.38:1
aconitase	gi|238014964	N33	Increase	2.05:1	1:4.53
Glyceraldehyde-3-phosphate dehydrogenase B, chloroplast	gi|108705994	N34	Increase	1:5.96	1:2.29
phosphoglucomutase, cytoplasmic 1	gi|162463106	N37	Increase	1.26:1	1:9.30
phosphoglycerate mutase	gi|293336560	N45	Increase	1:1.40	1:3.19
pyruvate dehydrogenase E1 component subunit beta	gi|195621752	N48	Increase	4.55:1	1:2.99
pyruvate dehydrogenase E1 beta subunit isoform 3	gi|162458813	N49	Increase	1.20:1	2.92:1
vacuolar ATP synthase catalytic subunit A(V-ATPase A subunit)	gi|195658441	N50	Increase	2.04:1	11.1:1
6-phosphogluconolctonase	gi|226493090	N58	Decrease	6.78:1	1:1.14
ATP synthase CF1 alpha subunit	gi|260677417	N62	Decrease	1:1.97	1:3.01
ATPase alpha subunit from chromosome 10 chloroplast	gi|19920165	N63	Decrease	2.96:1	1:1.28
ATP synthase CF1 alpha subunit	gi|50812525	N67	Decrease	1.27:1	1:4.11
Protein fate					
peptidylprolyl cis-trans isomerase	gi|226531796	N40	Qi319-96 Only	1:3.50	0:1
hsp70	gi|308081377	N53	Increase	4.02:1	3.06:1
cpn60 chaperonin family protein	gi|242090109	N56	Increase	1.69:1	4.71:1
chaperonin	gi|195623400	N72	Decrease	1.22:1	1.59:1
Protein synthesis					
elongation factor G	gi|242076604	N52	Increase	1.21:1	1:11.66
Transcription/cellularcomm-unication/signal transduction					
GTP binding protein	gi|242061356	N24	Increase	4.54:1	1:5.16
translation initiation factor	gi|162462542	N42	Increase	1:0	1.96:1
RNA binding protein	gi|212722236	N59	Decrease	1.97:1	2.81:1
Cell rescue, defense and virulence					
CBS domain protein	gi|226490863	N13	Increase	1:2.68	2.58:1
peptide methionine sulfoxide reductase	gi|293332177	N22	Increase	2.75:1	81.14:1
peptide methionine sulfoxide reductase	gi|226532399	N25	Increase	1.09:1	10.74:1
hydroxyproline-rich glycoprotein family protein	gi|226507242	N57	Decrease	1.19:1	1.89:1
Unknown					
predicted protein	gi|224157625	N60	Decrease	62.39:1	18.20:1
unknown protein	gi|18419782	N61	Qi319 only	2.00:1	1:0
hypothetical protein	gi|218185826	N65	Decrease	1:1.78	2.97:1
hypothetical protein VOLCADRAFT_104026	gi|302834273	N66	Decrease	1:3.02	31.68:1
clamp (CC)-tetratricopeptide repeat (TPR) proteins	gi|115446205	N68	Decrease	3.84:1	1:7.29
Secondary metabolism					
Caffeic acid O-methyltransferase	gi|33641714	N30	Increase	1:3.35	1:1.86
3-N-debenzoyl-2-deoxytaxol N-benzoyltransferase	gi|226500072	N31	Increase	1.81:1	1:2.71
dehydroquinate dehydratase	gi|255070969	N46	Increase	1.52:1	1:81.95
ornithine carbamoyltransferase	gi|255538702	N64	Decrease	84.38:1	30.71:1
ketol-acid reductoisomerase	gi|212722020	N70	Decrease	4.35:1	1:9.89

a: Name of protein identified by MALDI-TOF MS

b: Database accession numbers from NCBInr

c: Assigned spot number, as indicated in Fig. 2

d: “Increase” indicates significance at *P* < 0.05 and an increase in amount of at least 1.5-fold on the Qi319-96 gel under + P or –P conditions; “decrease”indicates significance at *P* < 0.05 and a decrease in amount of at least 1.5-fold on the Qi319-96 gel under + P or –P conditions compared withQi319

e: Specificity indicates the ratio of accumulation of a particular protein in leavesbetween Qi319-96 + P and Qi319-96 –P

f: Specificity indicates the ratio of accumulation of a particular protein in leavesbetween Qi319 + P and Qi319 – P

### Differentially accumulated proteins and their effects on photosynthesis in the low-phosphate-tolerant mutant and wild-type maize

**Fig. 3:**
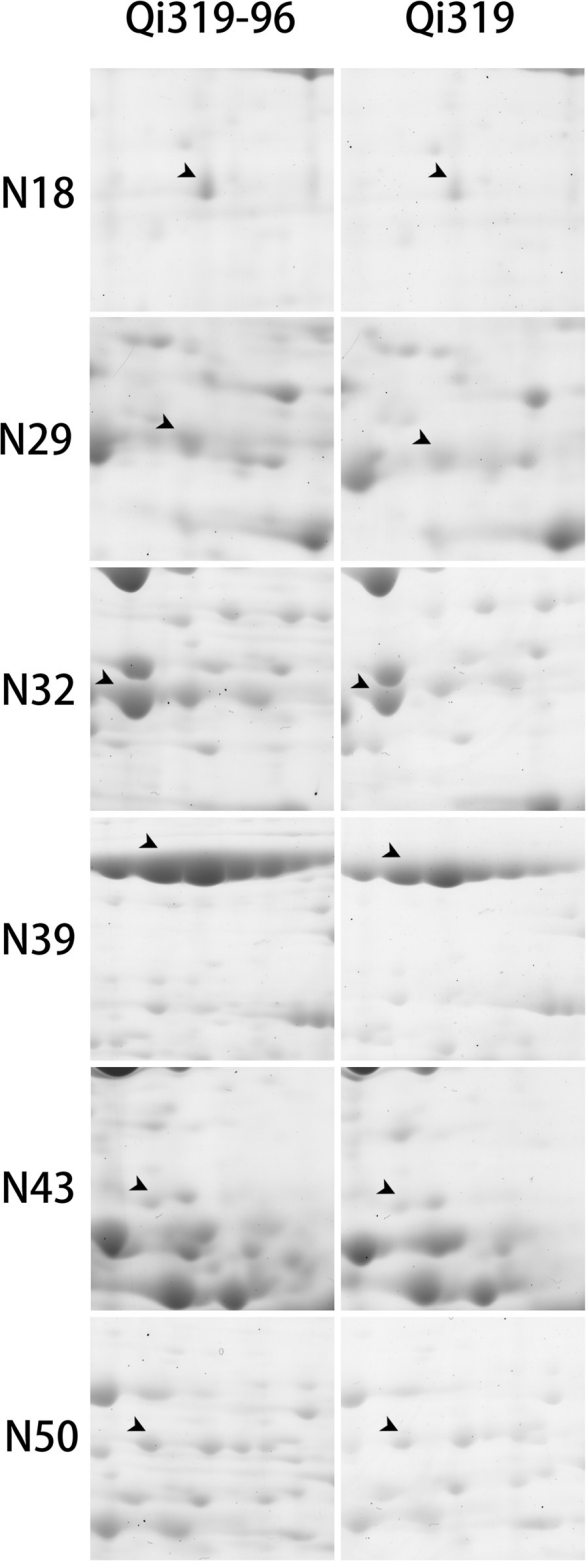
Comparison of 6 important protein between Qi319 and Qi319-96 under Pi-deficient condition. N18, uridine-5’-diphospho-sulfoquinovose (UDP-SQ) synthase; N29, fructose-bisphosphate (FBP) aldolase; N32, NADP-malic enzyme; N39, pyruvate orthophosphate dikinase; N43, delta-aminolevulinic acid dehydratase; N50, V-ATPase

The levels of several proteins that participate in photosynthesis were significantly different between the two genotypes under the two phosphate treatments. Under low phosphate conditions, the levels of RuBisCO (N3, N4, N5, N6, N7, N8, N9, N11, N12 and N14), NADP-malic enzyme (NADP-ME; N32 (Fig. 3), N35 and N36), pyruvate orthophosphate dikinase (PPDK; N38 and N39 (Fig. 3)), delta-aminolevulinic acid dehydratase (N43, Fig. 3), sucrose-phosphatase (N21), cytoplasm- phosphoglucomutase (PGM; N23 and N37), fructose-bisphosphate (FBP) aldolase (N26, N27, N28 and N29 (Fig. 3)), NADP-glyceraldehyde-3-phosphate dehydrogenase (NADP-GP3DH; N34), NADPH dihydroethidium (N19), plastoquinone-dehydrogenase (NADPH dehydrogenase; N55), and chlorophyll a/b binding protein (N15, N16 and N17) increased significantly compared with Qi319. To verify these differences, we performed several physiological and biochemical experiments. The RuBisCO, PGM, FBP aldolase, NADP-ME and PPDK activities were also higher in Qi319-96 than in Qi319 under low phosphate stress (Table 6), which was consistent with the two-dimensional gel electrophoresis (2-DE) results. FBP aldolase catalyzes the reaction between fructose-1,6-diphosphate and sedoheptulose-1,7-diphosphate during ribulose-1,5-bisphosphate (RuBP) regeneration. NADP-malic enzyme and PPDK play important roles in CO_2_ fixation in bundle sheath cells. The chlorophyll content in leaves from Qi319-96 was higher than from Qi319 by 50 % (Table 3), which may be related to the significant increase in delta-aminolevulinic acid dehydratase expression. Furthermore, the photosynthetic rate was 26.81 % higher in Qi319-96 than in Qi319 (Table 2). The proteome and physiological data showed that Qi319-96 has a higher photosynthetic rate due to its higher chlorophyll content, and the higher expression levels and activities of Calvin cycle and “CO_2_ pump” enzymes during phosphate stress.

**Table 6:** Influence of different phosphorus concentrations on the activities of several enzymes involved in photosynthesis

Inbred line	Pi treatment	RuBisCO (μmol CO_2_ mg pr^-1^ min^-1^)	PGM (μmol NADPH*mg^-1^ pr*min^-1^)	FBPaldose (μmol NADH/mg pr*min)	NADP-malic enzyme (μmol NADPH*mg^-1^ pr*min^-1^)	PPDK (μM AMP/mg Pr. min)
Qi319-96	+P	0.24 ± 0.03a	2.19 ± 0.12a	0.55 ± 0.02a	7.76 ± 0.24b	85.44 ± 2.71a
	–P	0.18 ± 0.08b	0.84 ± 0.04c	0.29 ± 0.08b	8.47 ± 0.22a	55.48 ± 2.02b
Qi319	+P	0.23 ± 0.05a	2.33 ± 0.12a	0.54 ± 0.04a	7.11 ± 0.05c	83.27 ± 1.28a
	–P	0.13 ± 0.06c	0.47 ± 0.01b	0.16 ± 0.01c	6.75 ± 0.11d	45.35 ± 1.59c

Three seedlings per bottle were cultured in phosphorus saturation solution (1000 μM KH_2_PO_4_) to the three-leaf stage, followed by low phosphate stress solution (5 μM KH_2_PO_4_) for an additional 25 days to the six–seven-leaf stage. Values represent the means of nine seedlings ± SD. Values with different letters within a row are significantly different (*P* < 0.05) by multiple comparison analysis

### Differentially accumulated proteins are involved in energy metabolism between the low-phosphate-tolerant mutant and wild-type maize

The levels of several proteins that participate in energy metabolism were significantly different between the two genotypes under the two phosphorus treatments. These proteins are involved in the tricarboxylic acid (TCA) cycle, the pentose phosphate pathway and glycolysis (EMP). Under the phosphorus deficiency treatment, NADP-non-phosphorylated glyceraldehyde-3-phosphate dehydrogenase (NADP-non-GAPDH; N1 and N2) accumulated, which may have allowed EMP to proceed smoothly under very low phosphate conditions [20]. Increases in the levels of aconitase (N33) and the pyruvate dehydrogenase complex (N48 and N49) in Qi319-96 may accelerate ATP synthesis through the TCA cycle in this line compared with in Qi319. The physiological data showed that the amount of ATP in maize leaves was 78.28 % higher in Qi319-96 than in Qi319 under low phosphate stress (Table 7).

**Table 7:** Influence of different phosphorus concentrations on ATP, SQDG, PG content and V-ATPase activity

Inbred line	Pi treatment	ATP (nmol*g^-1^ Fw)	SQDG (mol/%)	PG (mol/%)	V-ATPase (μM Pi mg^-1^ Pro.h)
Qi319-96	+P	395.22 ± 22.23a	9.25 ± 0.43a	8.22 ± 0.47a	8.36 ± 0.21a
	–P	134.53 ± 8.52b	13.45 ± 0.76b	6.12 ± 0.41b	12. 74 ± 0.32b
Qi319	+P	374.68 ± 17.45a	9.02 ± 0.24a	8.15 ± 0.55a	8.17 ± 0.11a
	–P	75.46 ± 4.47c	11.25 ± 0.88c	5.68 ± 0.33c	10.33 ± 0.23c

Three seedlings per bottle were cultured in phosphorus saturation solution (1000 μM KH_2_PO_4_) to the three-leaf stage, followed by low phosphate stress solution (5 μM KH_2_PO_4_) for an additional 25 days to the six–seven-leaf stage. Values represent the means of nine seedlings ± SD. Values with different letters within a row are significantly different (*P* < 0.05) by multiple comparison analysis

### Differentially accumulated proteins associated with increased phosphorus utilization between the low-phosphate-tolerant mutant and wild-type maize

Under low phosphate stress, the level of uridine-5’-diphospho-sulfoquinovose (UDP-SQ) synthase (N18, Fig. 3) increased significantly in Qi319-96 leaves compared with in Qi319 leaves. UDP-SQ synthase may increase the production of UDP-SQ, leading to an increase in available SQ, which is then used to produce SQDG. The increase in UDP-SQ synthase in Qi319-96 suggests that Qi319-96 may produce more SQDG than Qi319 under low phosphate stress. The accumulation of SQDG in the photo-membrane may displace phosphatidylglycerols (PG), which would accelerate the transformation of organic phosphorus and the utilization of internal phosphorus. The SQDG contents were 19.55 % higher in Qi319-96 than in Qi319, which is consistent with the UDP-SQ expression patterns for Qi319-96 and Qi319 (Table 7).

V-ATPase levels were not significantly different between Qi319-96 and Qi319 under the high phosphate treatment. However, under the low phosphate treatment, the increase in V-ATPase (N50, Fig. 3) levels was greater in Qi319-96 than in Qi319, suggesting that Qi319-96 might release phosphorus from the vacuole to increase the metabolic reaction rate in the cell, which would mitigate the symptoms caused by low phosphorus stress. The V-ATPase activity in the leaves was 23.33 % higher in Qi319-96 than in Qi319, which is consistent with the 2-DE results (Table 7).

### Other differentially accumulated proteins between the low-phosphate-tolerant mutant and wild-type maize

The abundance of some proteins, including molecular chaperones (M14, M15, M27, N22, N25 and N56), resistance-related proteins (M6, N13, N40 and N57), and proteins involved in protein synthesis (M18, N42 and N52), signal transduction (M7, N24 and N59), and secondary metabolism (N30, N46 and N64), were significantly different between Qi319-96 and Qi319 under different phosphate levels. Several proteins involved in protein folding and assembly, protein synthesis and mediating signal transduction accumulated differently between the two genotypes under different phosphate levels.

## Discussion

In this study, we applied comparative proteomics to gain insights into the phosphate-stress tolerance of Qi319-96 compared with Qi319. Compared with Qi319, the majority of the phosphate-stress-responsive proteins in Qi319-96 are involved in photosynthesis and internal phosphorus mobilization.

### Altered membrane lipid compositions, and increased V-ATPase activities and abundances in Qi319-96, increased the phosphorus use efficiency under low phosphate stress

To counteract the detrimental effects of phosphate stress, higher plants have evolved a series of mechanisms for maintaining internal phosphorus levels. An important adaptive strategy used by plants subjected to low phosphate conditions is to increase the internal phosphorus utilization efficiency by remodeling the lipid membrane [9]. One-third of the total organophosphate contents in plants is present as phospholipids. When plants are subjected to low phosphate stress, membrane phospholipids are replaced by non-phosphorus glycerolipids, which promotes the mobilization of organophosphates [10]. SQDG and PG are thought to be involved in maintaining phosphorus levels in the thylakoid membrane, and their contents are known to be regulated by the phosphorus level [21]. Under the low phosphate treatment, the expression of UDP-SQ synthase increased in Qi319-96 (compared with Qi319) to supply SQ polar groups for SQDG biosynthesis. Indeed, the physiological data showed that the SQDG content was higher in Qi319-96 than in Qi319 under phosphate deprivation, indicating that SQDG accumulates in the photo-membrane, which could then be used to supplement PG levels. These changes may accelerate organophosphate conversion and increase available phosphorus recycling during periods of low phosphate stress. There is a great interest in the ability of Qi319 to increase internal phosphorus utilization through lipid remodeling [19]. Compared with wild-type Qi319, Qi319-96 exhibited an increase in UDP-SQ expression in response to low phosphate stress, suggesting that UDP-SQ synthase plays a crucial role in maintaining an internal Pi balance in this maize mutant.

Phosphorus in the vacuole acts as a cushion under fluctuating external and internal phosphorus levels. Phosphorus may move from the vacuole to the cytoplasm and chloroplasts, thereby participating in metabolic reactions during phosphate stress [7]. Therefore, the vacuole plays a key role in maintaining cytoplasmic phosphorus homeostasis [22]. Phosphorus movement across the membrane depends on an electrochemical H^+^-gradient across the membrane, which is maintained by V-ATPase [2]. V-ATPase is more highly accumulated in Qi319-96 than in Qi319 during phosphate stress. The mutant may utilize the additional V-ATPase to intensify vacuolar-membrane proton transport, forming an electrochemical gradient across the membrane, and thus, increasing the energy supply for phosphorus transport across the membrane. This would significantly improve the tolerance of Qi319-96 to low phosphate stress.

Qi319-96 plants exhibited much better lipid composition remodeling and higher V-ATPase expression and activity levels than Qi319 plants, which could facilitate phosphorus utilization during periods of phosphate stress. Furthermore, these changes could be responsible for the increased Pi levels in Qi319-96 leaves compared with in Qi319 leaves.

### Increased expression of enzymes involved in the Calvin cycle and CO_2_ fixation may enhance photosynthesis in Qi319-96 compared with in Qi319

Phosphate deprivation has an adverse effect on plant photosynthesis [23]. The decline in photosynthetic efficiency in maize leaves under low phosphate stress is related to the reduced levels of proteins involved in CO_2_ enrichment, the Calvin cycle and the electron transport system. The reduced abundance of these proteins correlates well with the rates of photochemical reactions, CO_2_ assimilation, the Calvin cycle and RuBP regeneration [19]. Several enzymes that play key roles in photosynthesis, such as RuBisCO and 3-phosphoglyceric phosphokinase, are involved in plant responses to low phosphate conditions [24]. RuBisCO plays an important role in carbon fixation during the Calvin cycle [25]. Reduced RuBisCO activity is one of the non-stomatal factors that leads to a substantial decline in photosynthesis [26]. In the current study, RuBisCO was more highly expressed in Qi319-96 than in Qi319, and RuBisCO carboxylase activity was significantly higher in Qi319-96 than in Qi319, which may be related to the increased rate of photosynthesis in Qi319-96.

A reduction in RuBP regeneration has a considerable impact on photosynthesis. The rate of photosynthesis may decrease if RuBP regeneration declines [26]. Reductions in RuBP regeneration may be due to reduced ATP levels when plants are subjected to low phosphate conditions. Reduced ATP levels inhibit the reduction of 3-phosphoglycerate to triose phosphate through 3-phosphoglyceric phosphokinase, and they also inhibit the phosphorylation of ribulose-5-phosphate to RuBP through phosphoribulokinase [27, 28]. The increased accumulation of ATP in the leaves of Qi319-96 compared with Qi319 under low phosphate stress may be beneficial for RuBP regeneration. In addition, the reduced RuBP regeneration may be due to the reduced initial activities of several enzymes, such as NADP-GAPDH and FBP aldolase. RuBP regeneration is also affected by the increased amount of photosynthetic carbon that is diverted to starch formation and away from RuBP regeneration (RuBP is only found in chloroplasts) [29]. The higher level and activity of FBP aldolase in the chloroplasts of Qi319-96 versus Qi319 under phosphorus deprivation conditions may have led to a greater regeneration of RuBP in Qi319-96 compared with in Qi319.

Maize is a C4 plant with “CO_2_ pumps” that have a “floral hoop structure”. The “CO_2_ pump” mechanism in C4 plants increases the level of CO_2_ around RuBisCO, which may increase the carboxylation reaction rate and reduce the alternative fixation of O_2_. C4 photosynthesis is more efficient than C3 photosynthesis under low atmospheric CO_2_ conditions, which suggests that the “CO_2_ pump” is an important mechanism that leads to an increase in photosynthesis [30]. The accumulation and activity levels of NADP-ME and PPDK were higher in Qi319-96 than in Qi319 under phosphate deprivation conditions, which favored a greater capture rate and level of CO_2_ in vascular bundle sheath cells. Higher CO_2_ levels not only increase RuBisCO carboxylase activity, but they also suppress the oxidation of RuBisCO and reduce photorespiration. The higher CO_2_ level in Qi319-96 helped the plants increase carbon assimilation and counteract phosphate deprivation.

In conclusion, Qi319-96 had a higher rate of photosynthesis than Qi319 under low phosphate conditions, which could be attributed to its higher chlorophyll content and increased levels/activities of photosynthesis-related enzymes, such as RuBisCO, PPDK and NADP-ME.

## Conclusions

Qi319-96 had a significantly higher level of Pi than Qi319 under low-phosphate stress, which may be related to Qi319-96’s remodeled membrane lipids and the increase in V-ATPase activity, which releases phosphorus from plant organophosphorus to the cytoplasm. A physiological analysis showed that the carbon assimilation rate of Qi319-96 was significantly higher than that of Qi319 under low-phosphate stress, which may be owing to the increased accumulation of several photosynthesis-related in the mutant Qi319-96 compared with wild-type Qi319. Our results clearly indicated that the differences in increasing the internal P-use efficiency are the main reasons for the higher tolerance to low-P conditions in the mutant compared with the wild-type. At the same time, this study suggests that the inbred lines of maize with low-P tolerant traits could be obtained effectively through cellular engineering.

## Methods

### Plant growth and treatments

Maize mutant Qi319-96 with a low-P tolerance was obtained using cellular engineering technology. The immature embryo of maize inbred line Qi-319 were transferred to inducing medium to produce calli. Embryogenic calli were subcultured on subculture medium for 6 months, and then screened continuously on selecting medium without phosphate. After selection for 5 generations, we transferred the survival embryogenic calli to differentiation medium for the regeneration of plantlets (R_0_). The survival plants (R_0_) were transplanted to the field, and self-pollinated to harvest seeds (R_1_). Low-P tolerant R_1_ plants and their inbred progeny were selected by low phosphorus stress. After six generations, low-P tolerant inbred lines, including Qi319-96, were obtained [31, 32]. Seeds of the maize inbred line Qi319 and mutant Qi319-96 were sterilized with ethanol and HgCl_2_, and germinated in the dark at 28 °C. Three-day-old seedlings were grown in nutrient solution (+P, Pi-sufficient, 1,000 μM KH_2_PO_4_) for 15 days (to the three-leaf stage). Half of the seedlings were then cultured in low-phosphate nutrient solution (–P, Pi-deficient, 5 μM MKH_2_PO_4_). For the low-phosphate treatment, the 1,000 μM KH_2_PO_4_ in the + P nutrient solution was substituted with 1,000 μM KCl. The control nutrient solutions were the same as those used by Li et al. [31]. Seedlings were positioned in a completely randomized design in a greenhouse, and three batches of seedlings were cultured separately to provide biological replicates.

### Measuring the biomass, total phosphorus content, and Pi level

The maize seedlings were transferred to a phosphate-deficiency solution (5 μM KH_2_PO_4_), cultured for an additional 25 days (to the six- to seven-leaf stage), and washed with distilled water. The shoots and roots were weighed after drying at 80 °C to a constant weight. The phosphorus contents of the roots and shoots were determined according to Murphy and Riley [33]. The Pi level in the shoots was determined by measuring the molybdenum complex content, as described by Taussky and Shorr [34].

### Measuring ATP levels

Fresh samples (1 g) from the middle of the fourth leaves were boiled in 5 mL of MgSO_4_ for 15 min and centrifuged at 5,000 × *g* for 15 min at 4 °C. The supernatant was stored on ice until analyzed. The maize leaf ATP level was determined using the method described by Fan et al. [35].

### Measuring chlorophyll, sucrose, and starch contents

Fresh samples (0.1 g) were extracted in 80 % acetone, and chlorophyll levels were analyzed according to Arnon [36]. The sucrose and starch levels were assayed with resorcinol as previously described [19].

### Determining photosynthetic performance

To characterize photosynthetic performance in the maize plants, a portable photosynthesis system (LI-6400; LI-COR, Inc., Lincoln, NE, USA) was used to detect *P*n, ambient carbon dioxide (*C*o), and *C*i levels in the fourth expanded leaf of each sample. The *L*s value was then calculated using the following formula: *L*s = 1 – *C*i/*C*o. The photon flux density was kept at 800 μmol m^–2^ s^–1^ using an internal LED source, the temperature in the leaf chamber was maintained at 25 °C, and the relative humidity was 55–60 %. The CO_2_ level was approximately 360 μmol CO_2_ mol^–1^. All of the measurements were carried out between 09:30 am and 11:30 am.

### Enzyme activity assays

Fresh samples (1 g) were rapidly collected from the third leaf (from the bottom) of each plant and rapidly ground in 4 mL of buffer (0.1 mM Hepes-NaOH, pH 7.5, 50 mM MgCl_2_, 2 mM EDTA, 2 % polyvinylpyrrolidone, and 1 % β-mercaptoethanol) pre-cooled on ice. The homogenates were centrifuged at 15,000 × *g* for 20 min at 4 °C, and the supernatant was used for the enzyme assays [37]. PPDK was determined by assaying for NADH oxidation in a mixture containing 0.15 M Tris-HCl, 18 μM MgCl_2_, 30 μM dithiothreitol (DTT), 0.45 μM NADH, 3 μM phosphoenolpyruvate, 3 μM AMP, 3 μM sodium pyrophosphate, 6 units of lactic dehydrogenase, and an aliquot of leaf extract. The assays were initiated by adding 3 μM sodium pyrophosphate [37]. For the NADP-ME assay, an aliquot of leaf extract was added to a mixture containing 50 mM Hepes-KOH (pH 8.0), 5 mM DTT, and 0.5 mM NADP. The reaction was initiated by adding MgCl_2_ [37]. The mixture for the FBP aldolase assay contained 30 mM Hepes-KOH (pH 7.6), 10 mM FBP, 0.25 mM NADH, and 2–4 units mL^-1^ of alpha-glycerol-3-phosphate dehydrogenase and triose phosphate isomerase. The reaction was initiated by adding FBP [29]. PGM activity was determined after its reaction with NADP by measuring the change in absorbance at 340 nm. The reaction mixture contained 30 mM Hepes-KOH, 4 mM MgCl_2_, 0.5 mM NADP, and 2–4 units of glucose-6-phosphate dehydrogenase. The reaction was initiated by adding 1.2 mM glucose-1-phosphate [38]. The RuBisCO assay reaction mixture contained 50 mM Hepes-KOH (pH 8.0), 1 mM EDTA–2Na, 20 mM MgCl_2_, 25 mM DTT, 10 mM NaHCO_3_, 5 mM ATP, 0.15 mM NADH, 5 mM creatine phosphate, 0.6 mM RuBP, 10 units of phosphocreatine kinase, 10 units of glyceraldehyde-3-phosphate dehydrogenase, and 10 units of phosphoglycerate kinase. RuBisCO activity was determined by monitoring the absorbance change at 340 nm owing to the oxidation of NADH according to the method of Sawada et al. [39]. For the V-ATPase assay, vesicle membranes were isolated by sucrose density gradient ultracentrifugation according to Wang et al. [40].

### Lipid extraction, purification, and analysis

Fresh samples (0.5 g) were ground to a powder in liquid nitrogen and suspended in chloroform and methanol. The lipid was extracted and purified according to Blihg and Dyer [41]. The mixture was separated into individual lipids by two-dimensional thin-layer silica gel chromatography (G model, 10 cm × 10 cm). The first dimension was composed of acetone/methylbenzene/H_2_O_2_ (91:30:8 v/v/v), and the second dimension was composed of chloroform/methanol/isopropamide/ammonia (65:35:0.5:5 v/v/v/v). The thin-layer chromatography plates were sprayed with 0.01 % Primulin in acetone/water (3:2 v/v) and analyzed under a ultraviolet light (366 nm) to identify the locations of individual lipids. Spots corresponding to the lipid classes were removed and methylated. The lipid contents were determined using gas chromatography with heptadecanoic acid as an internal standard. The relative contents of individual lipids are presented as molar percentages (mol %) [42].

All physiological experimental data represent the means of three biological replicates ± SD. A significance analysis was performed using Duncan’s multiple range tests. All graphs were constructed using Sigma Plot 13.0.

### Protein sample preparation and 2-DE mapping

The fourth leaves from maize seedlings exhibiting phosphorus-stress symptoms were collected for protein extraction. Fresh samples (2 g) were ground to a powder in liquid nitrogen and combined with 20 mL of acetone containing 10 % TCA, 10 mM DTT, and 1 mM phenylmethylsulfonyl fluoride (PMSF). The mixture was precipitated at −20 °C overnight and then centrifuged at 19,000 × *g* for 20 min at 4 °C. The pellet was carefully washed twice in acetone containing 10 mM DTT and 1 mM PMSF to remove any pigment [43], and vacuum dried with a vacuum pump. The pellet was then dissolved in 2.5 mL of protein solubilization buffer [7 M urea, 2 M thiourea, 4 % CHAPS, 0.5 % v/v carrier ampholyte (pH 3–10), 10 mM DTT and 1 mM PMSF] for 2.5 h. The insoluble material was removed by centrifugation at 40,000 × *g* for 25 min. The protein level in the supernatant was measured using the Bradford assay and sub-sampled for 2-DE analysis [44].

The 2-DE was performed using pH 5–8 IPG strips (Bio-Rad, California, USA). Liquid rehydration buffer containing 1.2 mg of protein (7 M urea, 2 M thiourea, 4 % CHAPS, 1.5 % v/v carrier ampholyte, and 65 mM DTT) was used to hydrate the strips for 13 h using a GE Healthcare III (GE Healthcare, Buckinghamshire, United Kingdom). The voltage procedure was as follows: (1) grade voltage increased to 100 V for 30 min; (2) grade voltage increased to 250 V for 1 h; (3) step voltage increased to 1,000 V for 1 h; (4) step voltage increased to 5,000 V for 3 h; (5) grade voltage increased to 10,000 V for 6 h; and finally (6) step voltage increased to 10,000 V with the focus increased to 100 kVh. After isoelectric focusing, the IPG strips were equilibrated before sodium dodecyl sulfate polyacrylamide gel electrophoresis(SDS-PAGE) according to Yan et al. [45]. The strips were loaded onto 12 % denaturing acrylamide gels and sealed with 0.5 % agarose solution. The electrophoresis was carried out using a PROTEANII Ready Gel System (20 cm × 20 cm; Bio-Rad) at 10 mA gel^–1^ for 1 h and 25 mA gel^–1^ for 6 h. The gels were stained with Coomassie brilliant blue according to Katam et al. [46] and scanned using a GS-800 calibrated densitometer (Bio-Rad). The 2-DE experiment was carried out with three replications using independent samples. The images were analyzed using PDQuest software (version 7.2.0; Bio-Rad). After background subtraction and spot detection, the spots were matched and normalized using the total density in the gel image method.

The statistical significances of quantitative data were determined using the Student’s t-test (*n* = 3, *P* < 0.05) at a 95 % confidence level, and proteins with a 1.5-fold or more change at this confidence level were considered differentially accumulated.

### In-gel digestion and MALDI-TOF/MALDI-TOF-TOF MS analysis

Several protein spots were excised from gels and washed twice with distilled water to remove the redundant sodium dodecyl sulfate. The spots were destained in 25 mM NH_4_HCO_3_ [dissolved in 50 % acetonitrile (ACN)] and dehydrated in 100 % ACN. The protein spots were reduced, alkylated, and washed thoroughly, as described by Yan et al. [45]. The spots were then digested with 5–8 μL of trypsin (proteome grade trypsin; Sigma). The samples were dissolved in 40 mM ammonium bicarbonate and 9 % ACN at 20 ng mL^–1^ (pH 8.0) for 30 min at 4 °C. The redundant trypsin solutions were removed, and the gel pieces were dipped in 15 μL of 25 mM NH_4_HCO_3_ solution (pH 8.0) and incubated at 37 °C overnight. The supernatant was then transferred to new centrifuge tubes and the combined with 25 μL of solution containing 67 % ACN and 3.3 % trifluoroacetic acid (TFA). The two supernatant liquids were combined, dried under a vacuum, dissolved in 4–5 μL of 0.1 % TFA, and stored in 0.5-μL aliquots at −80 °C. Before analysis, the samples were mixed with 0.6 μL of 10 mg ml^–1^ w/v cyano-4-hydroxycinnamic acid in 0.1 % TFA/50 % ACN and dried on a metal plate. After air drying, the samples were subjected to MALDI-TOF MS and MALDI-TOF/TOF MS on a Bruker Ultraflex TOF/TOF controlled by the Flexcontrol 2.4 package using default parameters (Bruker Daltonics, Karlsruhe, Germany).

### Protein identification and database searching

After calibration and a monoisotopic peak analysis using GPS Explorer (Applied Biosystems 2006), the monoisotopic peak lists obtained were compared against the National Center for Biotechnology Information database using the MASCOT program (http://www.matrixscience.com), allowing one trypsin cleavage error. Carbamidomethylation of cysteine and oxidation of methionine were recognized as the fixed modifications, and pyro-glutamic acid formation of N-terminal glutamine was the variable modification. To obtain highly accurate identification results, the proteins had to fulfill the following criteria: (1) molecular weight search score > 73 (*P* < 0.05); (2) more than six peptides matched the theoretical result; (3) sequence coverage was greater than 15 %; and (4) the proteins had a peptide mass tolerance of at least 100 ppm. The search criteria for the MALDI-TOF-TOF/MS results were similar to the PMF criteria: (1) individual ions scores were > 43 (*P* < 0.05); (2) the peptide mass tolerance was 100 ppm; and (3) the fragment mass tolerance was at least 0.3 Da. The proteins identified using MALDI-TOF MS were categorized using the TAIR (http://www.arabidopsis.org/) and KEGG (http://www.genome.jp/kegg/) databases.

## Abbreviations

2-DE, two-dimensional gel electrophoresis; ACN, acetonitrile; EMP, glycolysis; IPG, immobilized pH gradient; MALDI-TOF MS, matrix-assisted laser desorption/ionization tandem time-of-flight mass spectrometry; PG, phosphatidylglycerol; Pi, inorganic phosphate; PMF, peptide mass finger printing; PMSF, phenylmethylsulfonyl fluoride; PPDK, pyruvate orthophosphate dikinase; RuBP, ribulose-1,5-bisphosphate; SQDG, sulfoquinovosyl diglyceride; TFA, trifluoroacetic acid; UDP-SQ, uridine-5’-diphospho-sulfoquinovose.

## Additional files

Additional file 1: Differentially accumulated proteins with similar functions present in the leaves of Qi319-96 and Qi319 under both + P and − P conditions. (DOC 2104 kb) Additional file 2: Identification of differentially accumulated proteins and sequences of the identified peptides. (XLS 203 kb) Additional file 3: MS/MS patterns of sequenced peptides. (DOC 1467 kb) Additional file 4: List of differentially expressed proteins in leaves of Qi319 and Qi319-96 seedlings under − P and + P conditions by MALDI-TOF-TOF/MS. (XLS 26 kb)
