# Supplementary material for: Physiological and comparative proteome analyses reveal low-phosphate tolerance and enhanced photosynthesis in a maize mutant owing to reinforced inorganic phosphate recycling
Source: BMC Plant Biol. 2016 Jun 8;16:129. doi: 10.1186/s12870-016-0825-1 (PMC4898391; doi:10.1186/s12870-016-0825-1)
Supplement: Additional file 1: — Differentially accumulated proteins with similar functions present in the leaves of Qi319-96 and Qi319 under both + P and − P conditions. (DOC 2104 kb) [file 12870_2016_825_MOESM1_ESM.doc]

**Appendix 1. Differentially accumulated proteins with similar function present in the leave of Qi319-96 and Qi-319 under both +P and −P conditions**

| Spot NO ^a^ | Accession No ^b^ | Specify ^c^ | Identification ^d^ | Mowse score ^e^ | PC ^f^ | Source ^g^ | Experimental  pI ^h^ | Theoretical  pI ^i^ | Experime-ntical  Mw ^j^ | Theoretical Mw ^k^ | sc% ^l^ | V%±SD^m^  KH_2_PO_4_  (µM)  5:1000 |
| --- | --- | --- | --- | --- | --- | --- | --- | --- | --- | --- | --- | --- |
| metabolism |  |  |  |  |  |  |  |  |  |  |  |  |
| M1 | gi|242088861 | 1:4.83 | Chlorophyll A-B binding protein | 82 | 7 | *Sorghum bicolor* | 5.06 | 5.14 | 42.6 | 28.1 | 30% |  |
| M2 | gi|194700378 | 1:2.55 | Chlorophyll A-B binding protein | 74 | 6 | *Zea mays* | 5.13 | 5.14 | 42.8 | 28.2 | 27% |  |
| M3 | gi|62738111 | 1:2.63 | Chain A, Pyruvate Phosphate Dikinase | 317 | 44 | *Zea mays* | 5.87 | 5.27 | 82.5 | 95.9 | 51% |  |
| M4 | gi|226498728 | 1:2.68 | malate dehydrogenase | 124 | 15 | *Zea mays* | 6.92 | 7.63 | 58.0 | 35.6 | 83% |  |
| M5 | gi|308081534 | 1:1.858 | Coproporphyrinogen III oxidase | 75 | 10 | *Zea maize* | 7.23 | 8.19 | 42.7 | 47. 4 | 25% |  |
| M9 | gi|194702698 | 1:3.07 | Triosephosphate isomerase | 106 | 16 | *Zea mays* | 6.73 | 6.14 | 58.2 | 32.7 | 57% |  |
| M10 | gi|194697898 | 8.91:1 | hydroxypyruvate reductase | 204 | 26 | *Zea mays* | 6.90 | 6.34 | 61.7 | 42.4 | 69% |  |
| M11 | gi|226491484 | 3.526:1 | thylakoid lumenal 19 KDa protein | 170 | 12 | Zea mays | 5.16 | 5.48 | 38.3 | 27.4 | 49% |  |
| M12 | gi|226506366 | 3.6:1 | sedoheptulose bisphosphatase1 | 143 | 20 | *Zea mays* | 5.12 | 6.08 | 58.6 | 42.3 | 39% |  |
| M13 | gi|194701566 | 1.87:1 | Cyanobacterial and plastid NDH-1 subunit M | 90 | 10 | *Zea mays* | 5.25 | 6.34 | 37.4 | 24.0 | 38% |  |
| M19 | gi|144583482 | 15.68:1 | ribulose-1,5-bisphosphate carboxylase/oxygenase large subunit | 228 | 25 | *Pogonatherum sp. Hodkinson 21* | 6.87 | 6.23 | 69.1 | 52.4 | 41% |  |
| M20 | gi|132061 | 5.92:1 | Ribulose bisphosphate carboxylase large chain | 94 | 11 | *Zea mays* | 6.62 | 6.13 | 39.7 | 53.1 | 34% |  |
| M21 | gi|11467200 | 2.45:1 | ribulose-1,5-bisphosphate carboxylase/oxygenase large subunit | 288 | 32 | *Zea mays* | 7.04 | 6.33 | 70.6 | 53.3 | 53% |  |
| M22 | gi|260677427 | 2.49:1 | ribulose-1,5-bisphosphate carboxylase/oxygenase large subunit | 115 | 17 | *Coix lacryma-jobi* | 5.73 | 6.13 | 42.9 | 53.4 | 39% |  |
| M23 | gi|195613254 | 1.98:1 | chlorophyll a-b binding protein 8 | 121 | 14 | *Zea mays* | 5.79 | 8.94 | 42.2 | 29.0 | 35% |  |
| M26 | gi|195619530 | 10.49:1 | oxygen-evolving enhancer protein 1 | 139 | 15 | *Zea mays* | 5.30 | 5.59 | 48.8 | 34.8 | 59% |  |
| M28 | gi|11467259 | 1:0 | NADH dehydrogenase subunit I | 134 | 11 | *Zea mays* | 6.86 | 6.42 | 42.3 | 21. 7 | 53% |  |
| N3 | gi|144583490 | 1:8.45 | ribulose-1,5-bisphosphate carboxylase/oxygenase large subunit | 275 | 30 | *Trachypogon spicatus* | 7.26 | 6.12 | 33.7 | 50.5 | 44% |  |
| N4 | gi|27448357 | 1:4.11 | ribulose-1,5-bisphosphate carboxylase/oxygenase large subunit | 75 | 9 | *Selaginella lepidophylla* | 6.13 | 6.14 | 29.0 | 50.2 | 24% |  |
| N5 | gi|290086785 | 1:6.76 | ribulose-1,5-bisphosphate carboxylase/oxygenase large subunit | 151 | 19 | *Karroochloa tenella* | 7.04 | 6.69 | 32.6 | 39.8 | 45% |  |
| N6 | gi|135991663 | 1:1.72 | ribulose-1,5-bisphosphate carboxylase/oxygenase large subunit | 261 | 32 | *Miscanthus capensis* | 7.06 | 6.46 | 33.9 | 49.9 | 53% |  |
| N7 | gi|3560858 | 1:11.69 | ribulose-1,5-bisphosphate carboxylase/oxygenase large subunit | 74 | 13 | *Vanilla aphylla* | 5.49 | 6.13 | 17.5 | 47.9 | 33% |  |
| N8 | gi|224382434 | 1:3.35 | ribulose-1,5-bisphosphate carboxylase/oxygenase large subunit | 92 | 8 | *Couepia guianensis* | 5.90 | 6.38 | 21.4 | 26.0 | 27% |  |
| N9 | gi|11467200 | 1:4.42 | ribulose-1,5-bisphosphate carboxylase/oxygenase large subunit | 297 | 35 | *Zea mays* | 6.88 | 6.33 | 55.9 | 53.3 | 55% |  |
| N11 | gi|144583486 | 1:4.62 | ribulose-1,5-bisphosphate carboxylase/oxygenase large subunit | 74 | 9 | *Sorghastrum nutans* | 6.94 | 6.34 | 21.5 | 52.9 | 16% |  |
| N12 | gi|261279200 | 1:12.37 | Ribulose bisphosphate carboxylase large chain | 89 | 7 | *Borojoa panamensis* | 6.16 | 5.52 | 21.8 | 19.2 | 29% |  |
| N14 | gi|164565217 | 1:3.21 | ribulose-1,5-bisphosphate carboxylase/ oxygenase large subunit | 149 | 14 | *Zizania latifolia* | 6.98 | 6.34 | 38.9 | 27.7 | 43% |  |
| N15 | gi|226496924 | 1:4.50 | chlorophyll a-b binding protein 8 | 73 | 6 | *Zea mays* | 6.92 | 6.82 | 43.2 | 16.8 | 38% |  |
| N16 | gi|223973225 | 1:2.48 | Chlorophyll A-B binding protein6A | 80 | 8 | *Zea mays* | 5.92 | 5.53 | 22.3 | 21.5 | 29% |  |
| N17 | gi|195613254 | 1:3.14 | chlorophyll a-b binding protein 8 | 104 | 9 | *Zea mays* | 6.05 | 8.94 | 32.6 | 28.9 | 34% |  |
| N18 | gi|238014584 | 1:2.92 | UDPsulfoquinovose synthase | 81 | 10 | *Zea mays* | 7.12 | 8.39 | 53.0 | 52.9 | 29% |  |
| N19 | gi|194704742 | 1:3.52 | NDH-dependent cyclic electron flow 1 NAD dependent epimerase | 159 | 15 | *Zea mays* | 6.45 | 6.95 | 44.3 | 38.3 | 51% |  |
| N21 | gi|194699500 | 1:2.90 | sucrose-phosphatase 1 | 131 | 17 | *Zea mays* | 6.31 | 5.64 | 54.2 | 47.6 | 44% |  |
| N32 | gi|162459265 | 1:1.80 | NADP-dependent malic enzyme,chloroplastic | 140 | 17 | *Zea mays* | 6.08 | 6.09 | 61.6 | 70.3 | 27% |  |
| N35 | gi|162459265 | 0:1 | NADP-dependent malic enzyme, chloroplastic precursor | 210 | 19 | *Zea mays* | 6.44 | 6.09 | 24.5 | 70.3 | 37% |  |
| N36 | gi|162459265 | 1:33.39 | NADP-dependent malic enzyme, chloroplastic | 179 | 27 | *Zea mays* | 5.92 | 6.09 | 61.9 | 70.3 | 34% |  |
| N38 | gi|285013667 | 1:3.28 | pyruvate orthophosphate dikinase | 234 | 37 | *Zea mays* | 5.9 | 5.71 | 25.4 | 106.0 | 39% |  |
| N39 | gi|168586 | 1:2.13 | pyruvate,orthophosphate dikinase | 295 | 47 | *Zea mays* | 6.09 | 5.71 | 75.3 | 103.4 | 44% |  |
| N41 | gi|219819651 | 0:1 | pyruvate orthophosphate dikinase | 121 | 14 | *Zea mays* | 5.54 | 5.55 | 74.9 | 100.3 | 16% |  |
| N43 | gi|226496321 | 1:2.41 | delta-aminolevulinic acid dehydratase | 139 | 20 | *Zea mays* | 5.79 | 6.04 | 48.1 | 46.4 | 48% |  |
| N44 | gi|255562878 | 1:3.92 | transferase, transferring glycosyl groups, putative | 75 | 11 | *Ricinus communis* | 5.75 | 7 | 51.7 | 71.6 | 16% |  |
| N51 | gi|226530882 | 1:3.96 | glucose-6-phosphate isomerase | 121 | 17 | *Zea mays* | 5.54 | 5.68 | 59.1 | 67.9 | 37% |  |
| N54 | gi|242060200 | 1:5.15 | Low PSII Accumulation 3 | 74 | 11 | *Sorghum bicolor* | 5.09 | 6.39 | 36.8 | 43.1 | 32% |  |
| N55 | gi|226497418 | 1:2.17 | subunit NDH-M of NAD(P)H:plastoquinone dehydrogenase | 91 | 9 | *Zea mays* | 5.53 | 6.34 | 21.4 | 23.9 | 50% |  |
| N69 | gi|293332983 | 2.2:1 | carbonate dehydratase | 118 | 13 | *Zea mays* | 7.08 | 6.60 | 58.6 | 29.7 | 53% |  |
| N71 | gi|226491484 | 2.01:1 | thylakoid lumenal 19 kDa protein | 139 | 14 | *Zea mays* | 5.43 | 5.48 | 22.2 | 27.4 | 57% |  |
| energy |  |  |  |  |  |  |  |  |  |  |  |  |
| M16 | gi|212274373 | 17.84:1 | Glyoxalase_I | 128 | 17 | *Zea mays* | 5.14 | 6.2 | 50.2 | 38.5 | 44% |  |
| M17 | gi|162461856 | 3.26:1 | glyceraldehyde-3-phosphate dehydrogenase A, chloroplastic | 157 | 17 | *Zea mays* | 5.22 | 7 | 46.3 | 41.3 | 56% |  |
| M29 | gi|22240 | 2.21:1 | GADPH (383 AA) | 205 | 22 | *Zea mays* | 6.87 | 7.21 | 59.4 | 41.3 | 69% |  |
| N1 | gi|194688752 | 1:7.38 | NADP+-dependent non-phosphorylating glyceraldehyde-3-phosphate dehydrogenase B | 87 | 10 | *Zea mays* | 6.19 | 5.95 | 72.2 | 47.7 | 26% |  |
| N2 | gi|194700892 | 1:4.99 | NADP+-dependent non-phosphorylating glyceraldehyde-3-phosphate dehydrogenase B | 148 | 16 | *Zea mays* | 7.35 | 6.8 | 57.5 | 53.9 | 36% |  |
| N10 | gi|162463282 | 1:2.17 | 6-phosphogluconate dehydrogenase family protein | 102 | 16 | *Zea mays* | 7.6 | 5.92 | 44.7 | 53.3 | 35% |  |
| N20 | gi|195613268 | 1:4.89 | alcohol dehydrogenase 2 | 118 | 17 | *Zea mays* | 6.45 | 5.72 | 47.6 | 41.8 | 39% |  |
| N23 | gi|162463106 | 1:2.86 | phosphoglucomutase, cytoplasmic 1 | 236 | 28 | *Zea mays* | 6.18 | 5.46 | 66.2 | 63.3 | 57% |  |
| N26 | gi|195622374 | 1:2.35 | fructose-bisphosphate aldolase | 182 | 16 | *Zea mays* | 5.78 | 5.39 | 40.8 | 40.5 | 43% |  |
| N27 | gi|223975775 | 1:5.13 | fructose-bisphosphate aldolase | 86 | 10 | *Zea mays* | 6.35 | 6.37 | 33.3 | 38.4 | 33% |  |
| N28 | gi|194690156 | 1:1.97 | fructose-bisphosphate aldolase | 75 | 10 | *Zea mays* | 7.59 | 7.52 | 45.3 | 39.0 | 46% |  |
| N29 | gi|195634659 | 1:1.65 | fructose-bisphosphate aldolase | 116 | 14 | *Zea mays* | 6.03 | 7.36 | 40.1 | 41.9 | 38% |  |
| N33 | gi|238014964 | 1:4.01 | aconitase | 123 | 13 | *Zea mays* | 5.99 | 6.76 | 54.4 | 55.4 | 37% |  |
| N34 | gi|108705994 | 1:2.33 | Glyceraldehyde-3-phosphate dehydrogenase B, chloroplast | 203 | 23 | *Zea mays* | 6.03 | 5.95 | 45.6 | 47.7 | 46% |  |
| N37 | gi|162463106 | 1:3.39 | phosphoglucomutase, cytoplasmic 1 | 86 | 12 | *Zea mays* | 6.04 | 5.46 | 65.7 | 63.3 | 27% |  |
| N45 | gi|293336560 | 1:6.03 | phosphoglycerate mutase | 74 | 9 | *Zea mays* | 5.83 | 5.47 | 63.4 | 60.4 | 23% |  |
| N48 | gi|195621752 | 1:1.94 | pyruvate dehydrogenase E1 component subunit beta | 81 | 14 | *Zea mays* | 5.66 | 5.46 | 42.9 | 40.2 | 39% |  |
| N49 | gi|162458813 | 1:2.48 | pyruvate dehydrogenase E1 beta subunit isoform 3 | 105 | 15 | *Zea mays* | 5.59 | 5.46 | 42.6 | 40.2 | 31% |  |
| N50 | gi|195658441 | 1:2.74 | vacuolar ATP synthase catalytic subunit | 252 | 30 | *Zea mays* | 5.97 | 5.3 | 66.1 | 68.7 | 54% |  |
| N58 | gi|226493090 | 2.21:1 | 6-phosphogluconolctonase | 89 | 8 | *Zea mays* | 5.49 | 5.08 | 31.8 | 28.9 | 35% |  |
| N62 | gi|260677417 | 2.86:1 | ATP synthase CF1 alpha subunit | 270 | 31 | *Coix lacryma-jobi* | 6.28 | 6.03 | 61.3 | 55.7 | 57% |  |
| N63 | gi|19920165 | 6.38:1 | ATPase alpha subunit from chromosome 10 chloroplast | 243 | 20 | *Oryza sativa Japonica Group* | 6.31 | 5.88 | 60.8 | 55.8 | 39% |  |
| N67 | gi|50812525 | 5.96:1 | ATP synthase CF1 alpha subunit | 299 | 31 | Saccharum officinarum | 6.25 | 5.87 | 60.9 | 55.7 | 52% |  |
| Protein fate |  |  |  |  |  |  |  |  |  |  |  |  |
| M6 | gi|226491656 | 1:2.745 | peptidyl-prolyl cis-trans isomerase | 89 | 10 | Zea mays | 6.95 | 9.28 | 35.1 | 26.4 | 37% |  |
| M14 | gi|195623400 | 8.49:1 | chaperonin | 93 | 8 | *Zea mays* | 5.18 | 8.67 | 41.8 | 25.6 | 52% |  |
| M15 | gi|194688414 | 68.56:1 | CPN10 | 78 | 6 | *Zea mays* | 5.19 | 8.49 | 42.0 | 25.8 | 39% |  |
| M24 | gi|168041407 | 1.66:1 | Pep nsin-like aspartate proteases | 76 | 14 | *Physcomitrella patens subsp. patens* | 5.75 | 5.67 | 44.8 | 78.6 | 25% |  |
| M27 | gi|226532399 | 9.56:1 | peptide methionine sulfoxide reductase | 95 | 8 | *Zea mays* | 6.15 | 5.85 | 37.9 | 20.8 | 47% |  |
| N40 | gi|226531796 | 0:1 | peptidylprolyl cis- trans isomerase | 74 | 6 | *Zea mays* | 7.66 | 8.8 | 19.5 | 16.4 | 37% |  |
| N53 | gi|308081377 | 1:13.30 | hsp70 | 172 | 28 | *Zea mays* | 5.56 | 5.08 | 67.1 | 71.2 | 46% |  |
| N56 | gi|242090109 | 1:2.48 | cpn60 chaperonin family protein | 108 | 16 | *Sorghum bicolor* | 5.23 | 5.07 | 59.5 | 60.9 | 40% |  |
| N72 | gi|195623400 | 4.72: 1 | chaperonin | 146 | 14 | *Zea mays* | 5.14 | 8.67 | 24.8 | 25.6 | 72% |  |
|  |  |  |  |  |  |  |  |  |  |  |  |  |
| protein synthesis |  |  |  |  |  |  |  |  |  |  |  |  |
| M18 | gi|22942613 | 2.02:1 | ribonucleoprotein | 94 | 12 | *Zea mays* | 5.4 | 5.75 | 41.6 | 18.1 | 65% |  |
| N52 | gi|242076604 | 1:3.32 | elongation factor G | 201 | 32 | *Sorghum bicolor* | 5.56 | 5.42 | 71.6 | 85.3 | 48% |  |
| Transcription/cellular communication /signal transduction |  |  |  |  |  |  |  |  |  |  |  |  |
| M7 | gi|195609654 | 1:4.28 | glycine-rich RNA-binding protein 2 | 73 | 7 | Zea mays | 6.76 | 6.1 | 26.9 | 15.5 | 57% |  |
| N24 | gi|242061356 | 1:10.16 | GTP-binding protein | 82 | 9 | *Sorghum bicolor* | 6.18 | 6.79 | 71.7 | 72.5 | 22% |  |
| N42 | gi|162462542 | 1:2.16 | translation initiation factor | 166 | 21 | *Zea mays* | 5.84 | 5.38 | 51.5 | 47.3 | 48% |  |
| N59 | gi|212722236 | 14.6:1 | RNA-binding protein | 97 | 14 | *Zea mays* | 7.98 | 8.89 | 39.2 | 41.4 | 38% |  |
| Cell rescue, defense and virulence |  |  |  |  |  |  |  |  |  |  |  |  |
| N13 | gi|226490863 | 1:1.93 | CBS domain protein | 119 | 14 | *Zea mays* | 7.64 | 9.35 | 18.9 | 22.5 | 79% |  |
| N22 | gi|293332177 | 1:7.09 | peptide methionine sulfoxide reductase | 159 | 14 | *Zea mays* | 6.32 | 5.85 | 22.1 | 20.8 | 58% |  |
| N25 | gi|226532399 | 1:1.83 | peptide methionine sulfoxide reductase | 89 | 7 | *Zea mays* | 6.04 | 5.85 | 21.5 | 20.8 | 43% |  |
| N57 | gi|226507242 | 12.05:1 | hydroxyproline-rich glycoprotein family protein | 178 | 20 | *Zea mays* | 7.14 | 6.3 | 41.8 | 38.8 | 60% |  |
| unknown |  |  |  |  |  |  |  |  |  |  |  |  |
| M8 | gi|242086601 | 1:2.61 | hypothetical protein SORBIDRAFT_09g001130 | 74 | 8 | *Sorghum bicolor* | 6.46 | 6.99 | 46.9 | 31.9 | 27% |  |
| M25 | gi|162462462 | 5.71:1 | LOC542632 | 96 | 8 | *Zea mays* | 5.67 | 5.39 | 45.9 | 25.3 | 47% |  |
| N60 | gi|224157625 | 25.45:1 | predicted protein | 72 | 6 | *Populus trichocarpa* | 5.51 | 9.3 | 37.1 | 14.9 | 45% |  |
| N61 | gi|18419782 | 1:0 | unknown protein | 81 | 9 | *Arabidopsis thaliana* | 5.29 | 5.61 | 36.4 | 27.5 | 38% |  |
| N65 | gi|218185826 | 2.43:1 | hypothetical protein | 74 | 10 | *Oryza sativa Japonica Group* | 5.32 | 9.2 | 31.3 | 24.1 | 50% |  |
| N66 | gi|302834273 | 2.01:1 | hypothetical protein VOLCADRAFT_104026 | 76 | 33 | *Volvox carteri f. nagariensis* | 5.39 | 6.29 | 34.5 | 35.6 | 11% |  |
| N68 | gi|115446205 | 9.15:1 | clamp (CC)-tetratricopeptide repeat (TPR) proteins | 81 | 12 | *Oryza sativa Japonica Group* | 7.97 | 6.34 | 47.3 | 48.8 | 27% |  |
| second metabolism |  |  |  |  |  |  |  |  |  |  |  |  |
| N30 | gi|33641714 | 1:2.21 | Caffeic acid O-methyltransferase | 112 | 13 | *Zea mays* | 6.04 | 5.48 | 42.2 | 39.2 | 40% |  |
| N31 | gi|226500072 | 1:2.10 | 3-N-debenzoyl-2-deoxytaxol N-benzoyltransferase | 117 | 14 | *Zea mays* | 5.97 | 5.43 | 50.6 | 46.5 | 39% |  |
| N46 | gi|255070969 | 1:22.21 | dehydroquinate dehydratase | 74 | 7 | *Micromonas sp. RCC299* | 5.51 | 5.66 | 16.3 | 55.8 | 17% |  |
| N64 | gi|255538702 | 2.61:1 | ornithine carbamoyltransferase | 87 | 13 | *Ricinus communis* | 5.32 | 7.2 | 33.1 | 41.7 | 33% |  |
| N70 | gi|212722020 | 4.89:1 | ketol-acid reductoisomerase | 160 | 24 | *Zea mays* | 6.36 | 6.31 | 60.3 | 63.3 | 42% |  |

a: Assigned spot numbers as indicated in Figure 2

b: Database accession numbers from NCBInr

c: Specificity indicates the ratio of accumulation of a particular protein from leaf between Qi319 and Qi319-96 under different phosphorus concentration.

d: The name of the proteins identified by MALDI-TOF-MS

e: The Mascot score obtained after searching against the NCBInr database

f: Number of peptides identified for predicted protein

g: The plant species that the peptides matched from.

h: Experimental pI identified proteins. The experimental values were calculated using PDQuest Software (Version 7.2)

i: Theoretical pI of identified proteins. Theoretical values were retrieved from the protein database.

j: Experimental mass (KDa) of identified proteins. The experimental values were calculated using PDQuest Software (Version 7.2)

k: Theoretical mass (KDa) of identified proteins. Theoretical values were retrieved from the protein database

l: The amino acid sequence coverage for the identified proteins

m: The mean values of protein spot volumes relative to total volume of all the spots. Two phosphorus treatments (5µM and 1000 µM) were performed. Error bars indicate (standard deviationr, SD).
