# Supplementary material for: Physiological and comparative proteome analyses reveal low-phosphate tolerance and enhanced photosynthesis in a maize mutant owing to reinforced inorganic phosphate recycling
Source: BMC Plant Biol. 2016 Jun 8;16:129. doi: 10.1186/s12870-016-0825-1 (PMC4898391; doi:10.1186/s12870-016-0825-1)

**Appendix 3. The MS/MS patterns of sequenced petides**

**Spot number:** M12

**Protein name:** Sedoheptulose-1, 7-bisphosphatase, chloroplastic

**gi|1173347**

**Sequenced peptide:** MFSPGNLR

FEETLYGSSR


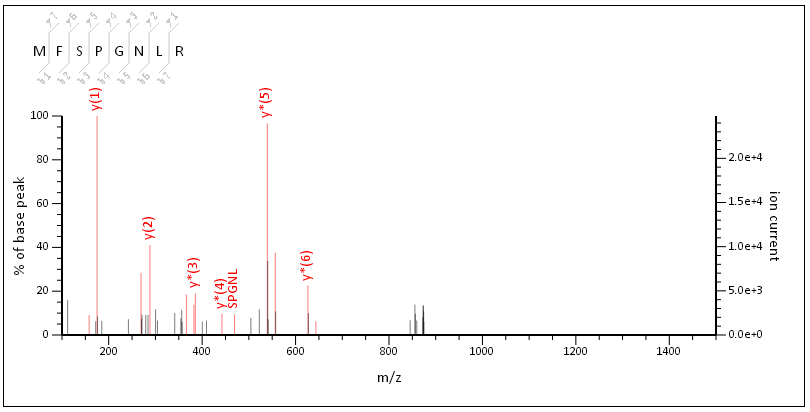
LLFEALEYSHVCK

**
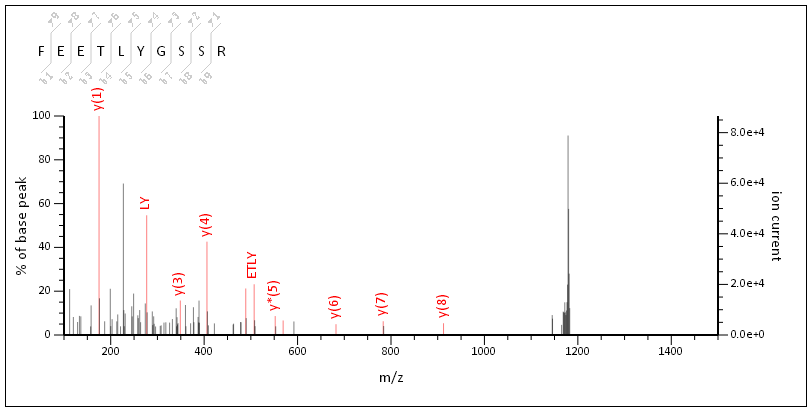
**

**
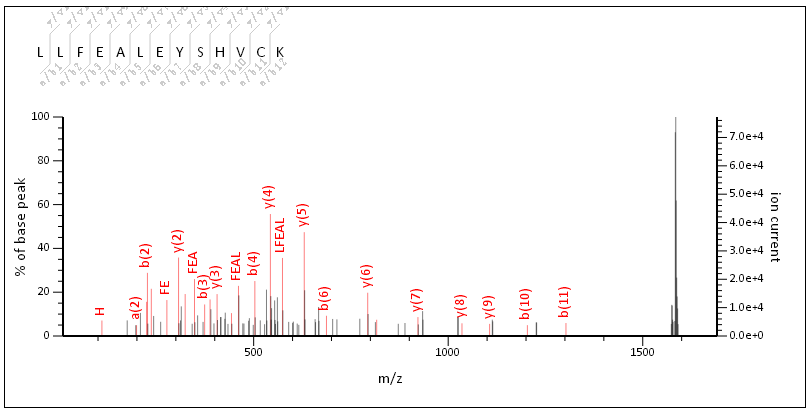
**

**Spot number:** M19

**Protein name:** Ribulose bisphosphate carboxylase large chain

**gi|131979**

**Sequenced peptide:** VALEACVKAR

TFQGPPHGIQVER

GGLDFTKDDENVNSQPFMR

**
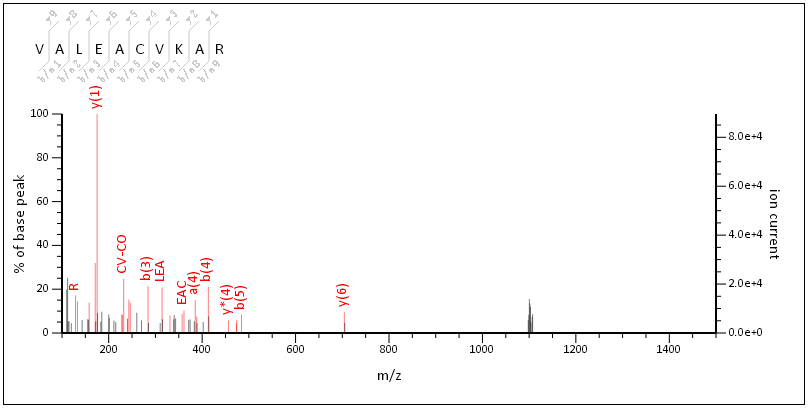
**

**
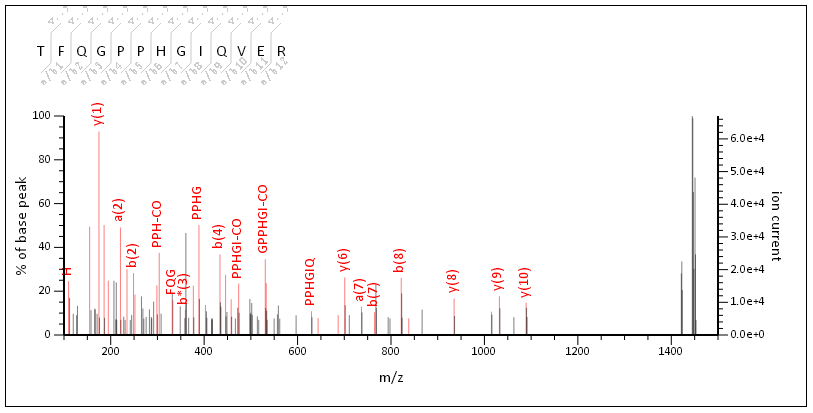
**

**
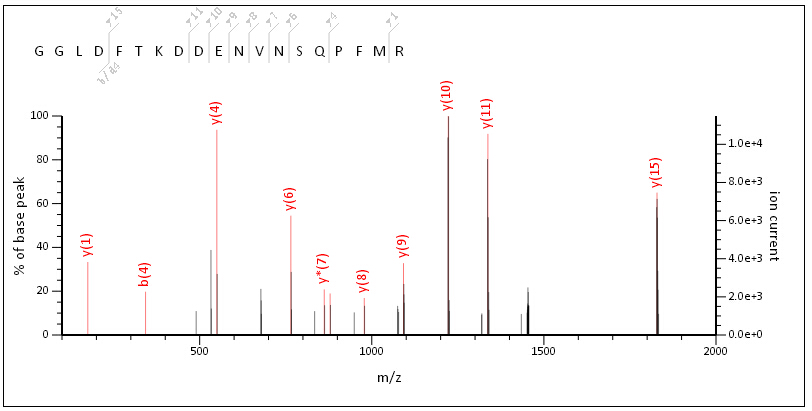
**

**Spot number:** M28

**Protein name:** NADH dehydrogenase subunit I

**gi|11467259**

**Sequenced peptide:** LPITIHYPYEK

HELNYNQIALSR

LPISIMGDYTIQTIR


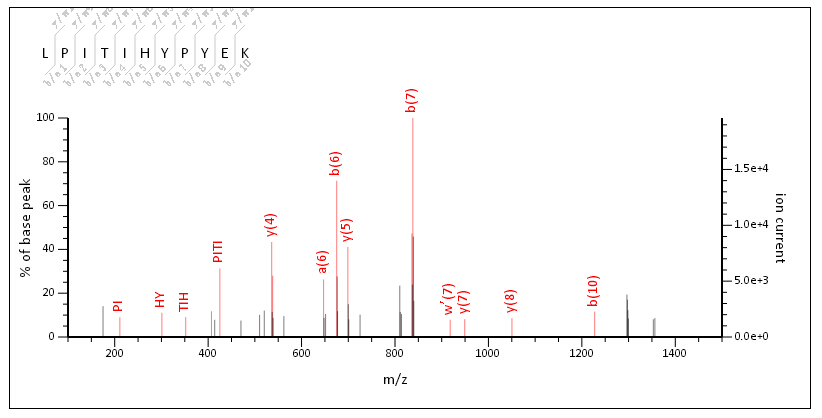


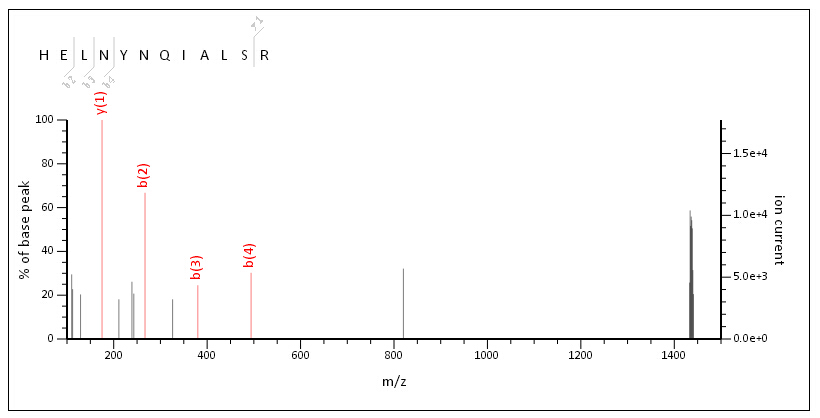


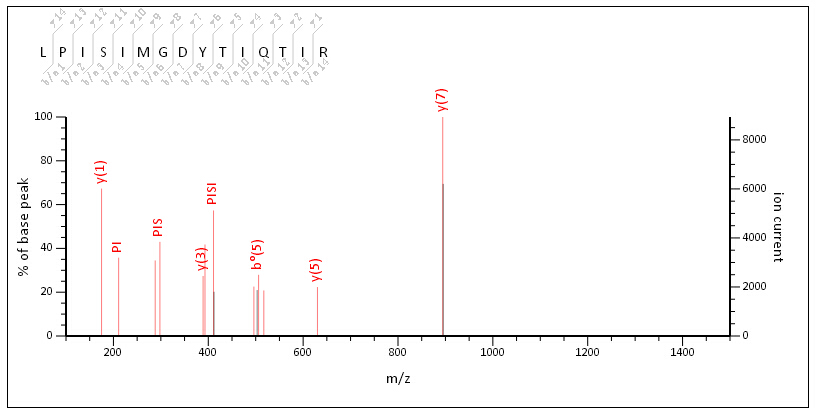


**Spot number:** N14

**Protein name:** ribulose-1,5-bisphosphate carboxylase/oxygenase large subunit

**gi|168335**

**Sequenced peptide:** DTDILAAFR

VALEACIKGR

TFQGPPHGIQVER


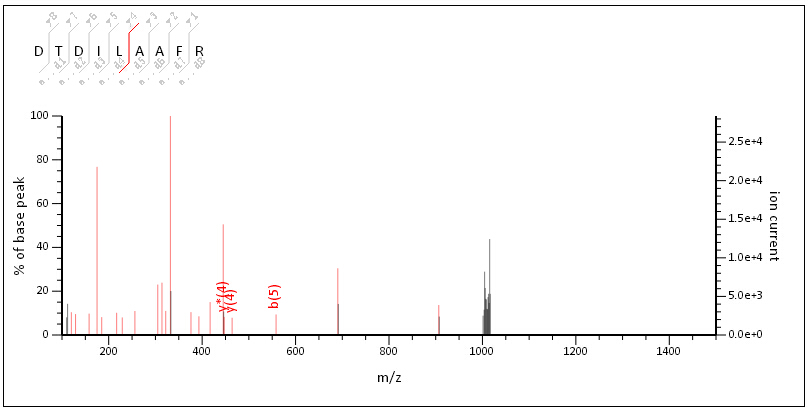


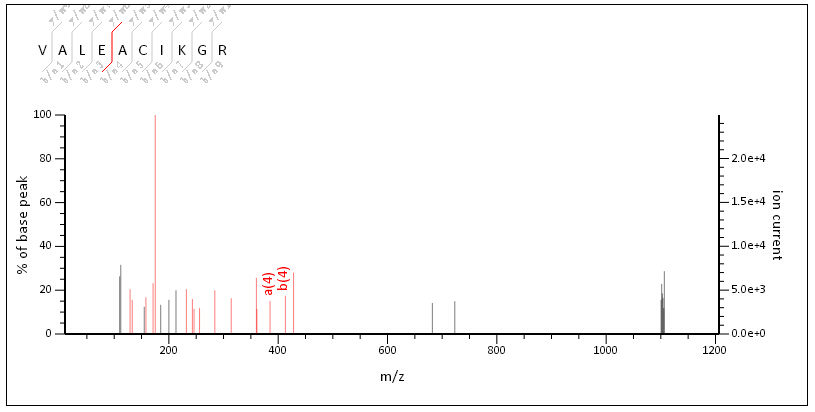


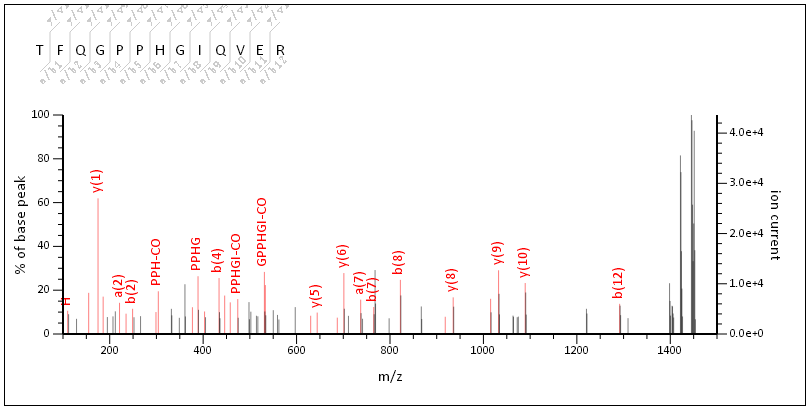


**Spot number:** N17

**Protein name:** chlorophyll a-b binding protein 8

**gi|195613254**

**Sequenced peptide:** WLAYGEVINGR

YAMLGAVGAIAPEIFGK

FLAGSGDPSYPGGPLFNPLGFGK


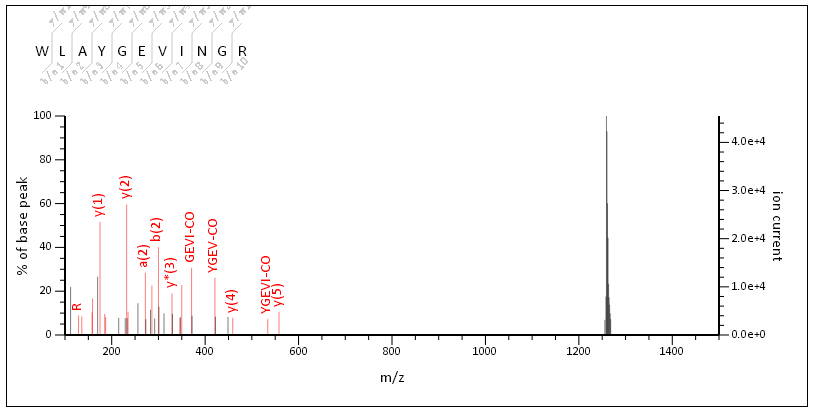


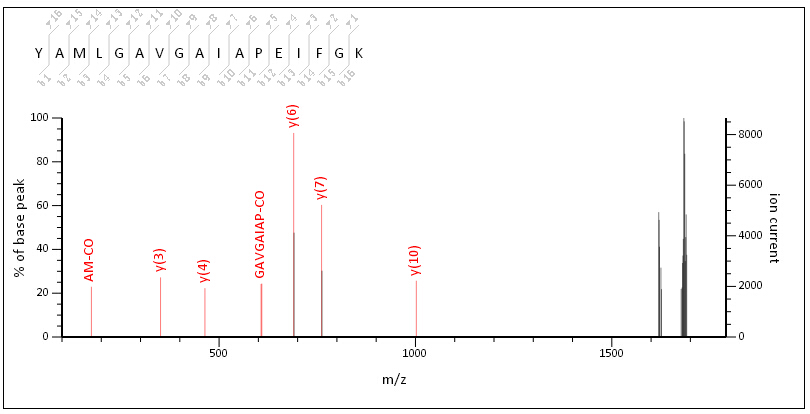


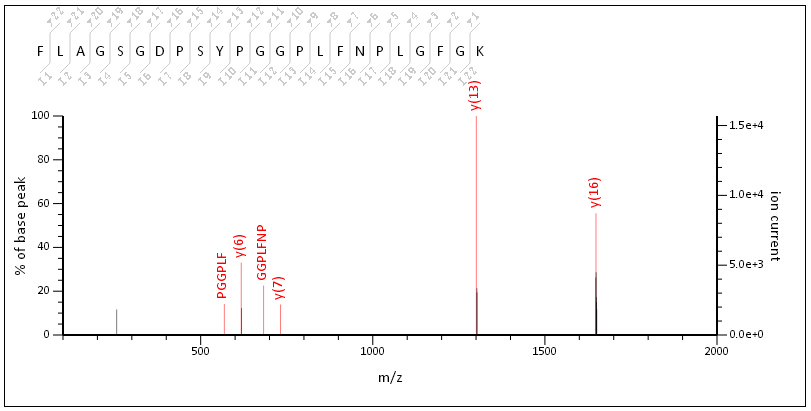


**Spot number:** N26

**Protein name:** fructose-bisphosphate aldolase

**gi|195622374**

**Sequenced peptide:** EAAYYQAGAR

ATPEQVAEYTLR


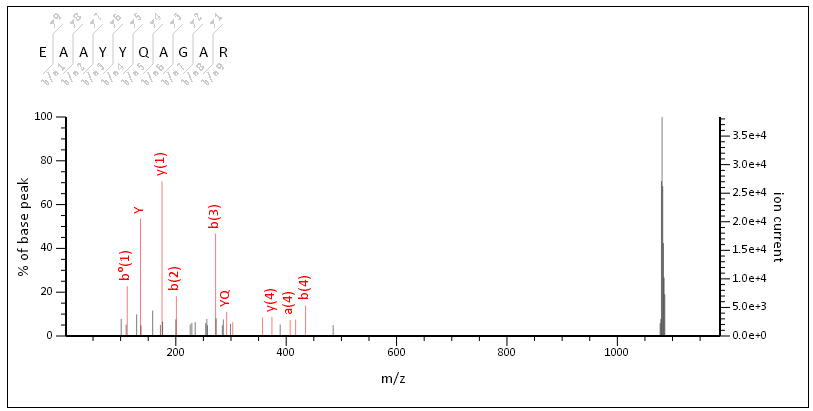


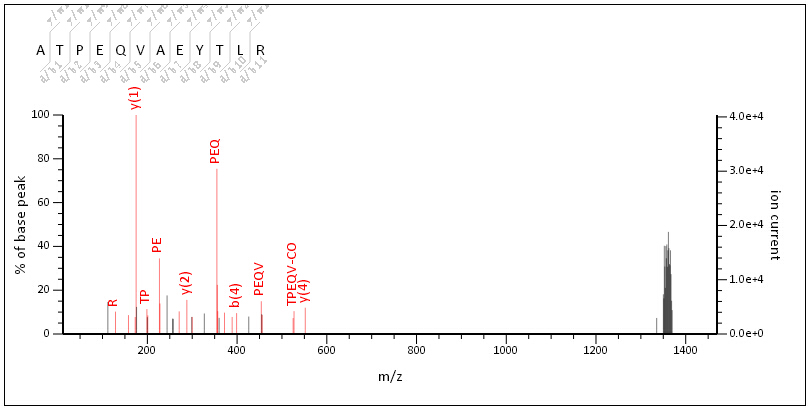


**Spot number:** N36

**Protein name:** NADP-malic enzyme

**gi|30575690**

**Sequenced peptide:** LLNDEFYIGLR

NIQVICVTDGER


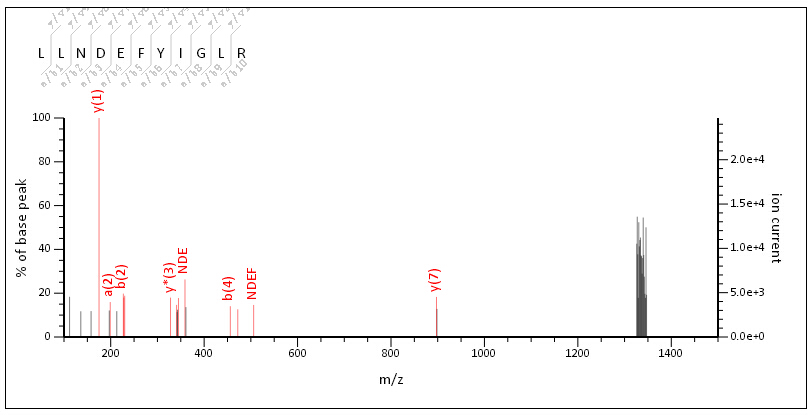


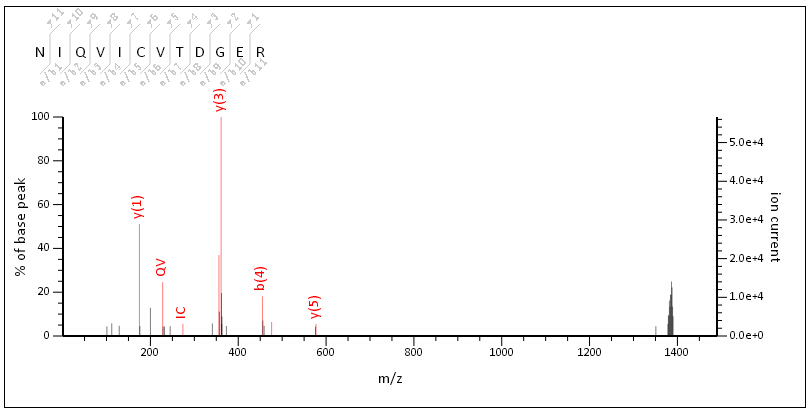


**Spot number:** N48

**Protein name:** pyruvate dehydrogenase E1 beta subunit isoform 2

**gi|162458637**

**Sequenced peptide:** SNYMSAGQISVPIVFR

IAGADVPMPYAANLER


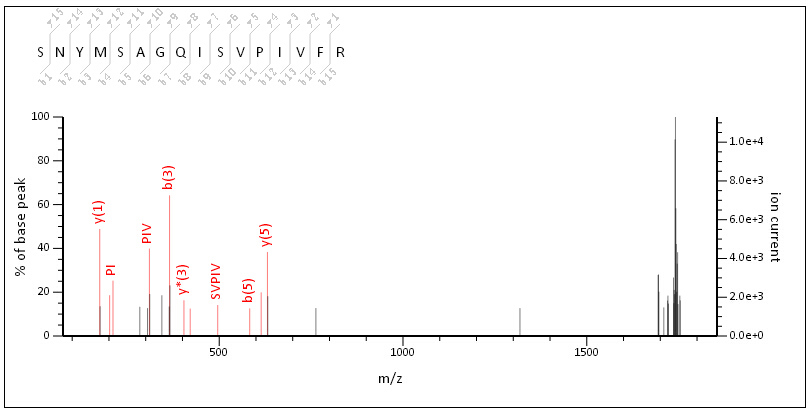


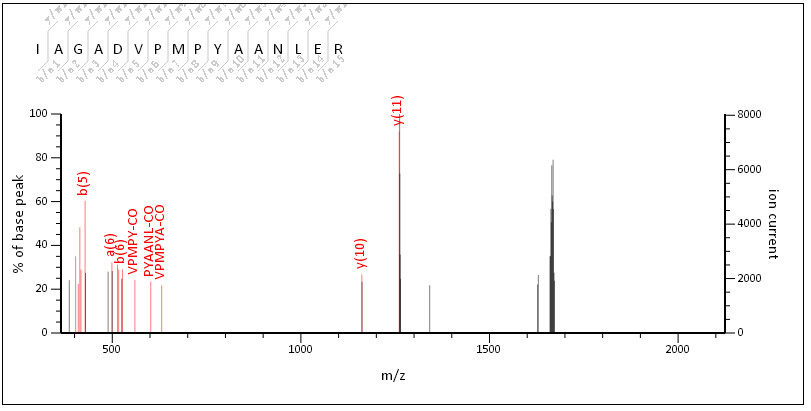

Supplement: Additional file 3: — MS/MS patterns of sequenced peptides. (DOC 1467 kb) [file 12870_2016_825_MOESM3_ESM.doc]
